# Supplementary material for: Intratumoral injection of IL-12-encoding mRNA targeted to CSFR1 and PD-L1 exerts potent anti-tumor effects without substantial systemic exposure
Source: Mol Ther Nucleic Acids. 2023 Jul 19;33:599–616. doi: 10.1016/j.omtn.2023.07.020 (PMC10450355; doi:10.1016/j.omtn.2023.07.020)
Supplement: Document S1. Figures S1–S11 [file mmc1.pdf]

OMTN, Volume *33*

Supplemental information

Intratumoral injection of IL-12-encoding mRNA targeted to CSFR1 and PD-L1 exerts potent anti-tumor effects without substantial systemic exposure

Claudia Augusta Di Trani, Assunta Cirella, Leire Arrizabalaga, Maite Alvarez, Ángela Bella, Myriam Fernandez-Sendin, Joan Salvador Russo-Cabrera, Celia Gomar, Nuria Ardaiz, Alvaro Teijeira, Elixabet Bolaños, José González-Gomariz, Itziar Otano, Fernando Aranda, Belén Palencia, Aina Segués, Shuyu Huang, Sander M.J. van Duijnhoven, Andrea van Elsas, Ignacio Melero, and Pedro Berraondo

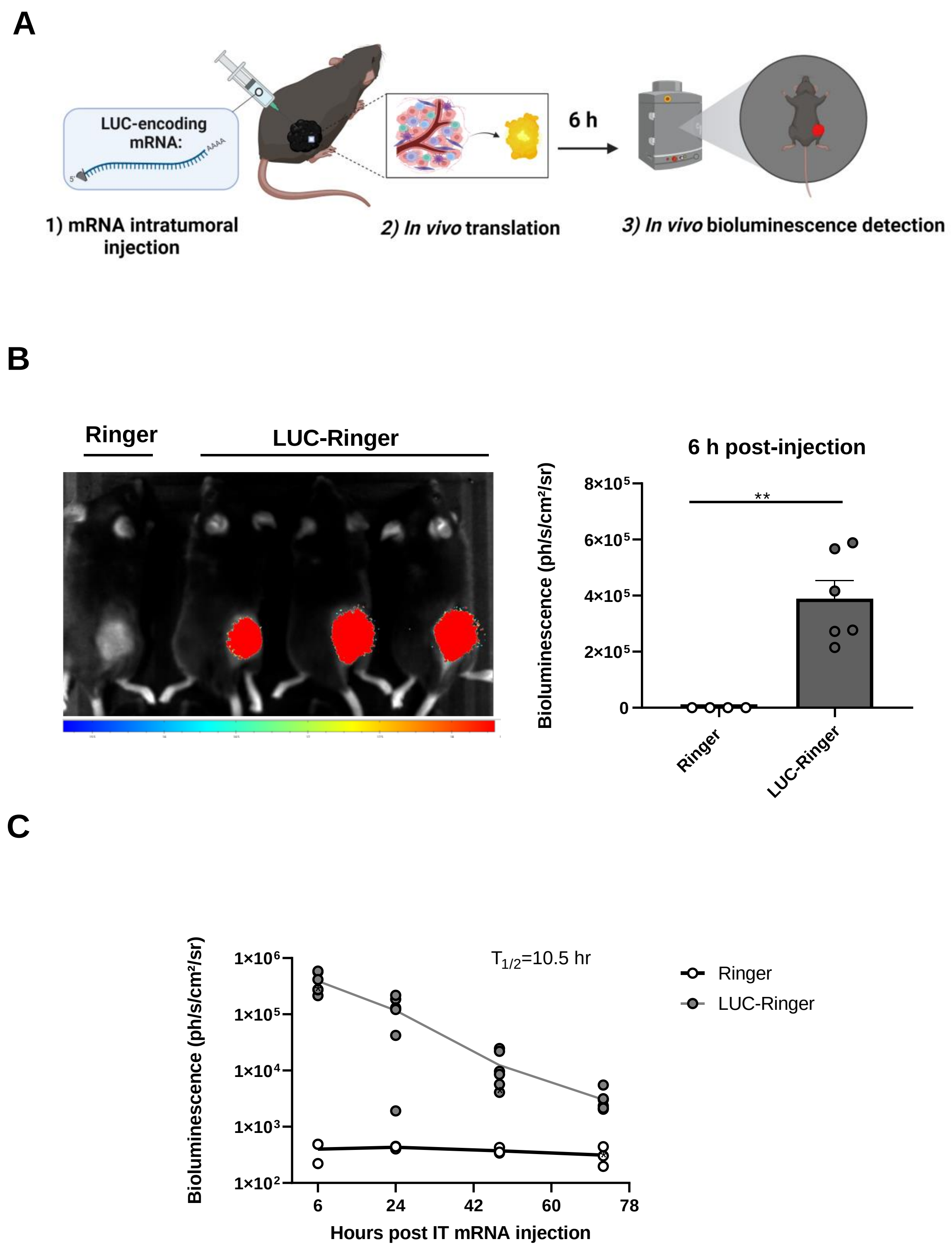

Figure S1. Intratumoral naked luciferase mRNA delivery leads to high expression in the tumor niche. A. Schematic representation of the experimental procedure. B16-OVA cells (0.5 x 10^6) were inoculated subcutaneously into the right flank of C57BL/6 mice (n=4-

6). Twelve days post-tumor inoculation, 10 µg of mRNA encoding luciferase were delivered intratumorally in 50 µl of Ringer's lactate solution. **B.** *In vivo* bioluminescence measured 6 h post-mRNA injection. Representative mice from each group are shown. The graph on the right includes the quantification of all mice in each experimental group. **C.** Bioluminescence follow-up. Expression half-life was determined by fitting it to a one-phase decay equation. Data are given as mean ± SEM, and statistical significance was determined with two-tailed unpaired t-test (Mann-Whitney) for panel B (**$p < 0.01$).

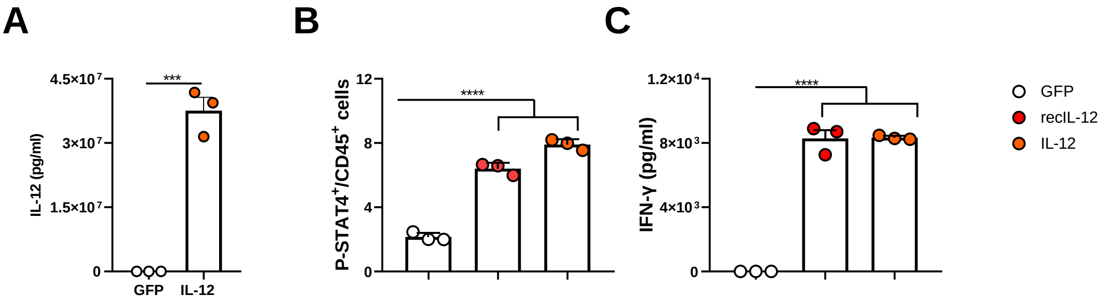

Figure S2. The mRNA-encoded scIL-12 is correctly secreted and bioactive *in vitro*. A. B16-OVA cells were transfected with mRNA encoding GFP or IL-12 formulated with the TransIT-mRNA Transfection kit. Supernatants were collected 72 h post-transfection and assayed for IL-12p70 detection by ELISA. **B.** Murine splenocytes were preactivated O/N using plate-bound anti-CD3 (1 µg/ml) and soluble anti-CD28 (1 µg/ml). The day after, 3 x 10^6 preactivated splenocytes were incubated for 1 h at 37 °C with the conditioned media from mRNA-transfected B16-OVA cells, containing 400 ng of IL-12, as previously assessed by the IL-12p70 ELISA sandwich assay. As a positive control, 400 ng of recombinant IL-12 were used and the supernatant of cells transfected with GFP-mRNA was employed as a negative control. Then, cells were washed and the pellet was resuspended in Cytofix buffer for 15 mins at 37 °C. After extensive washing, cells were permeabilized using Perm Buffer III for 30 mins at 4 °C and then stained with anti-CD45-PerCP and anti-P-STAT4-PE antibody for 45 minutes at RT. The percentage of $CD45^+$ cells with phosphorylated STAT-4 are shown. **C.** Murine splenocytes were preactivated in an anti-CD3-coated plate O/N. Then, they were treated with the supernatants collected 72 h after mRNA transfection of B16-OVA cells or with 50 ng of recombinant IL-12 as a positive control. Forty-eight hours after incubation, the splenocyte culture supernatants were analyzed for IFN-γ production by ELISA. Data are shown as mean ± SEM. Statistical significance was determined with one-way ANOVA followed by Dunnett's multiple comparison tests (***$p < 0.001$, ****$p < 0.0001$).

A

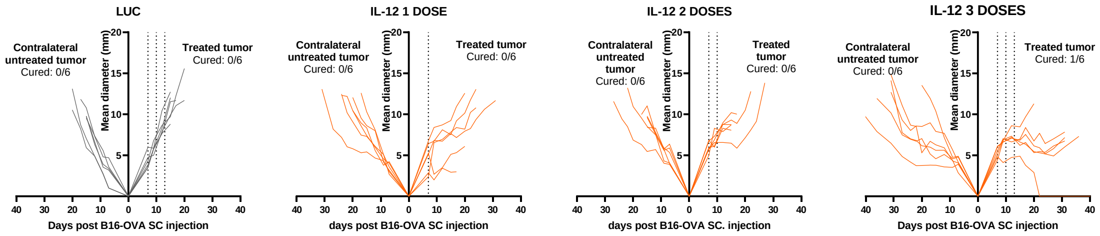

B

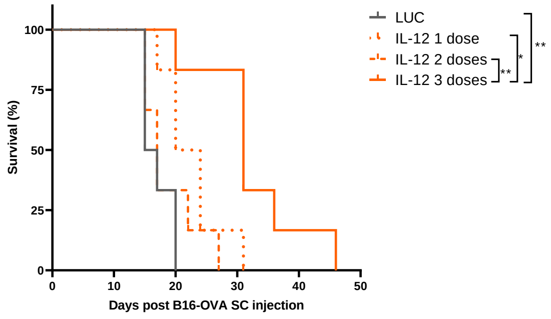

Figure S3. Three injections of IL-12-mRNA outperform a single or two doses in terms of antitumor efficacy. A. C57BL/6 mice (n=6) were injected with 0.5 x 10^6 B16-OVA cells subcutaneously in the right flank and 0.15 x 10^6 in the left flank. At the indicated time (dashed lines), mRNAs encoding LUC and IL-12 (0.5 µg/dose) were delivered intratumorally in 50 µl of Ringer's lactate solution. The one dose group was treated at day 7. The 2 dose-group was treated at day 7 and 10, and the 3 dose-groups (LUC and IL-12) were treated at day 7, 10 and 13. Individual follow-up of tumor sizes with the fraction of cured tumors is presented. **B.** Survival follow-up is shown and the data were analyzed by Log-rank (Mantel-Cox) tests (*$p < 0.05$, **$p < 0.01$).

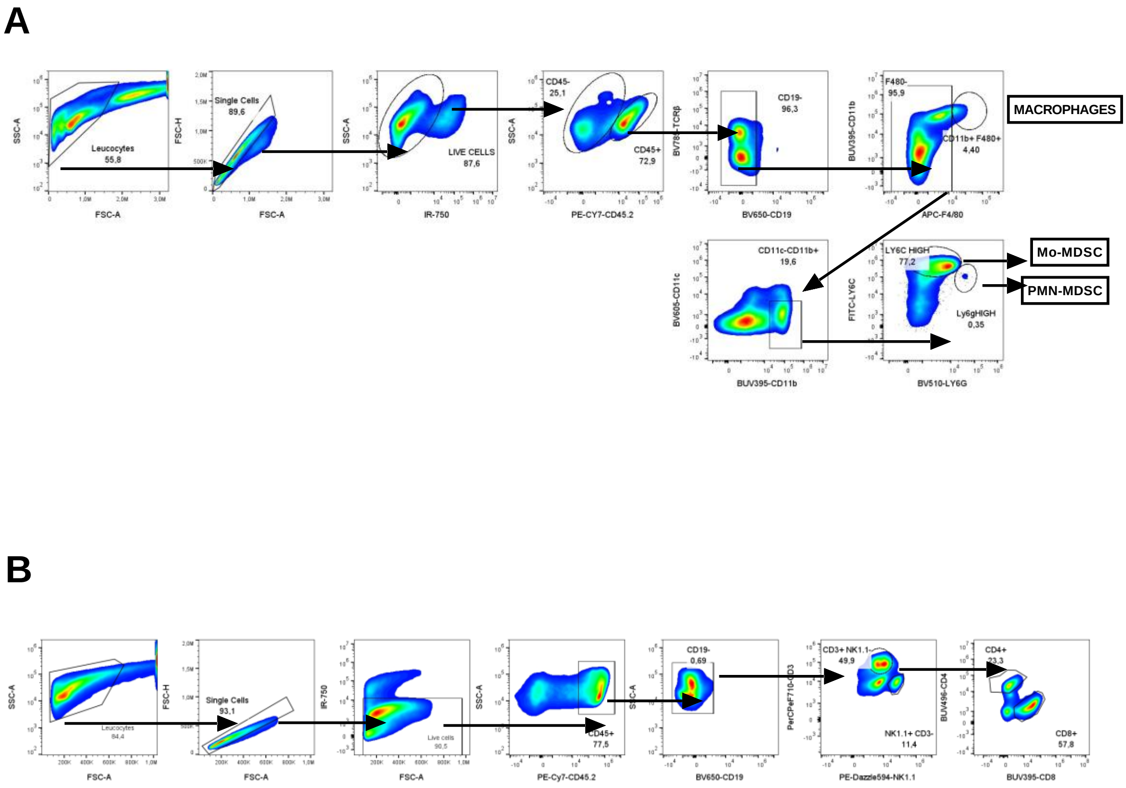

Figure S4. Gating strategy of multi-color flow cytometry analyses. A. Gating strategy employed in flow cytometry analyses to classify myeloid cell populations in Figs. 3-4-7. **B.** Gating strategy employed in flow cytometry analyses to classify lymphoid cell populations in Fig. 4 and Fig. S6.

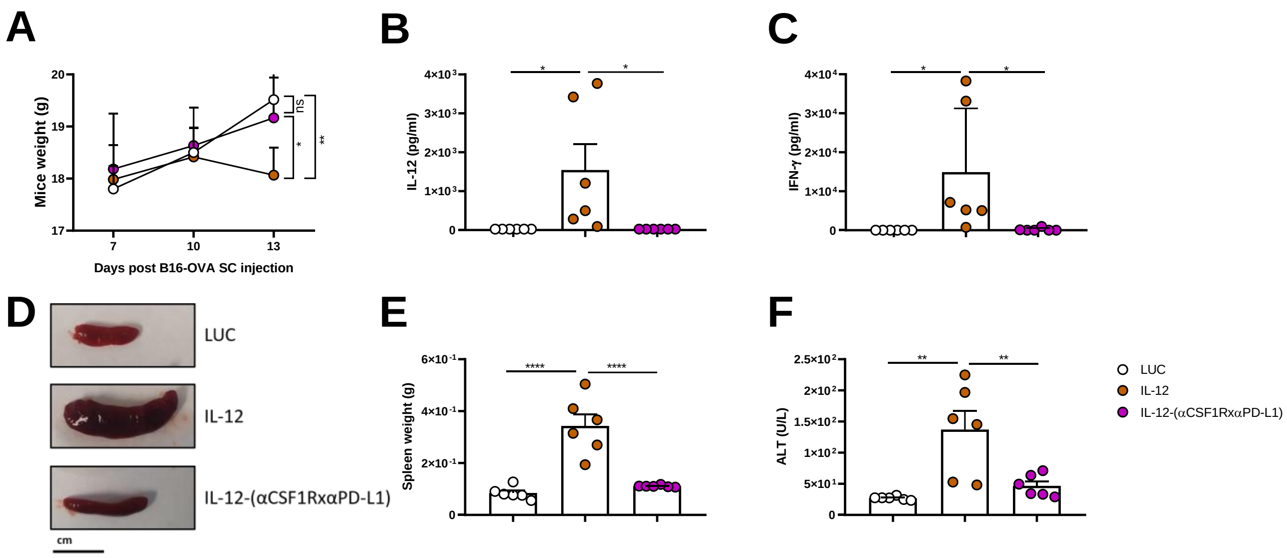

Figure S5. Mice treated with the mRNA encoding the IL-12-diabody are protected from systemic toxicity observed following the intratumoral administration of non-chimerized IL-12-mRNA. C57BL/6 mice were injected with 0.5 x 10^6 B16-OVA cells subcutaneously in both flanks (n=6/group). On days 7, 10, and 13 after tumor inoculation, each implanted tumor was injected with 10 µg of each indicated mRNA. **A.** Total body weight (g) measured before each dose injection. Statistical comparison of data collected on day 13. **B-F.** Twenty-four hours after the third dose, serum was collected from the mice for subsequent analyses, and mice were sacrificed. **B.** IL-12p70 and **C.** IFN-γ systemic levels were assessed in mice sera by commercial ELISA assays. **D.** Representative image of spleens for each condition. **E.** Quantification of spleen weights. **F.** Alanine transaminase in sera of mice measured with an automatic biochemical analyzer Cobas c-311 (Roche Diagnostics). Data are shown as mean ± SEM. Statistical significance was determined with one-way ANOVA followed by Tukey's multiple comparison tests in panels A, B, C, E, F (*p < 0.05, **p < 0.01, ****p < 0.0001).

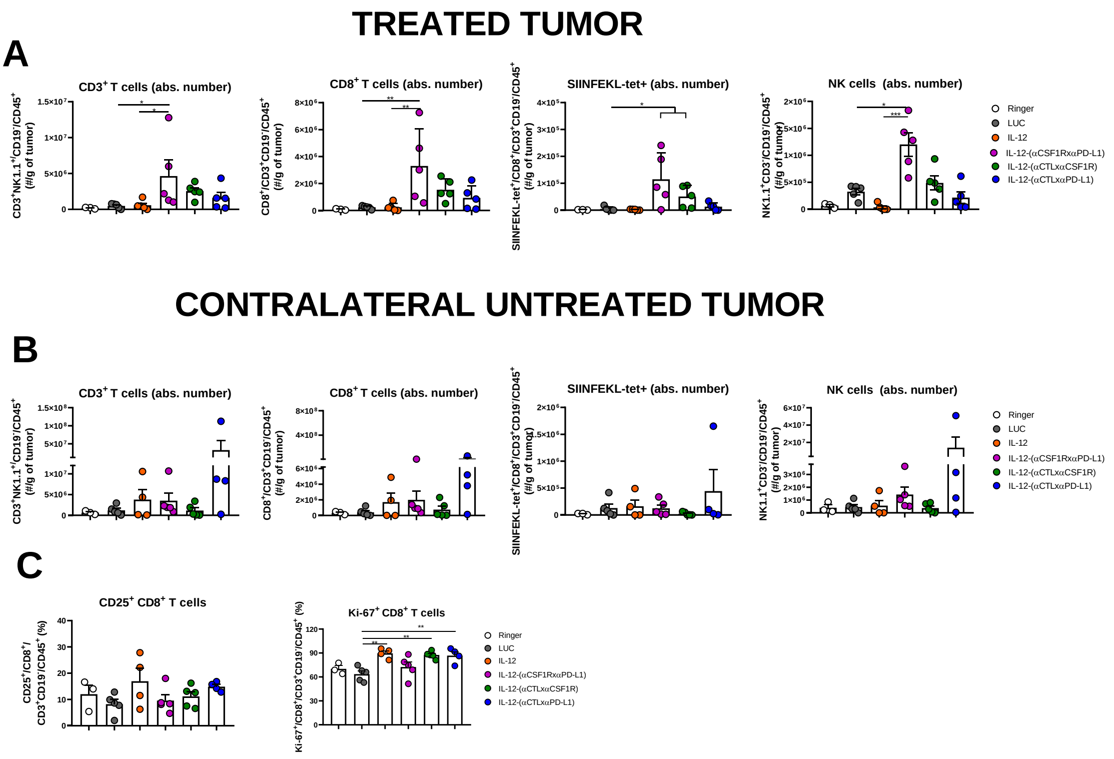

Figure S6. Lymphocyte infiltration in treated and uninjected lesions. A. B16-OVA-bearing mice (n=3-5) received two doses of the indicated mRNAs on day 7 and day 10 after tumor injection and were sacrificed 4 days after the last treatment (0.5 µg of IL-12-encoding mRNA and 10 µg of the mRNAs encoding the diabodies were used per each dose). Immune cells of the excised tumors were phenotypically characterized by multi-color flow cytometry analysis. **A.** Absolute numbers of different immune populations in treated tumors and **B.** contralateral untreated tumors are shown. **C.** The activation and proliferation states of $CD8^+$ T cells in contralateral tumors were evaluated. Data are shown as mean ± SEM. Statistical significance was determined with one-way ANOVA followed by Sidak's multiple comparisons tests (*$p<0.05$, **$p<0.001$, ***$p < 0.001$).

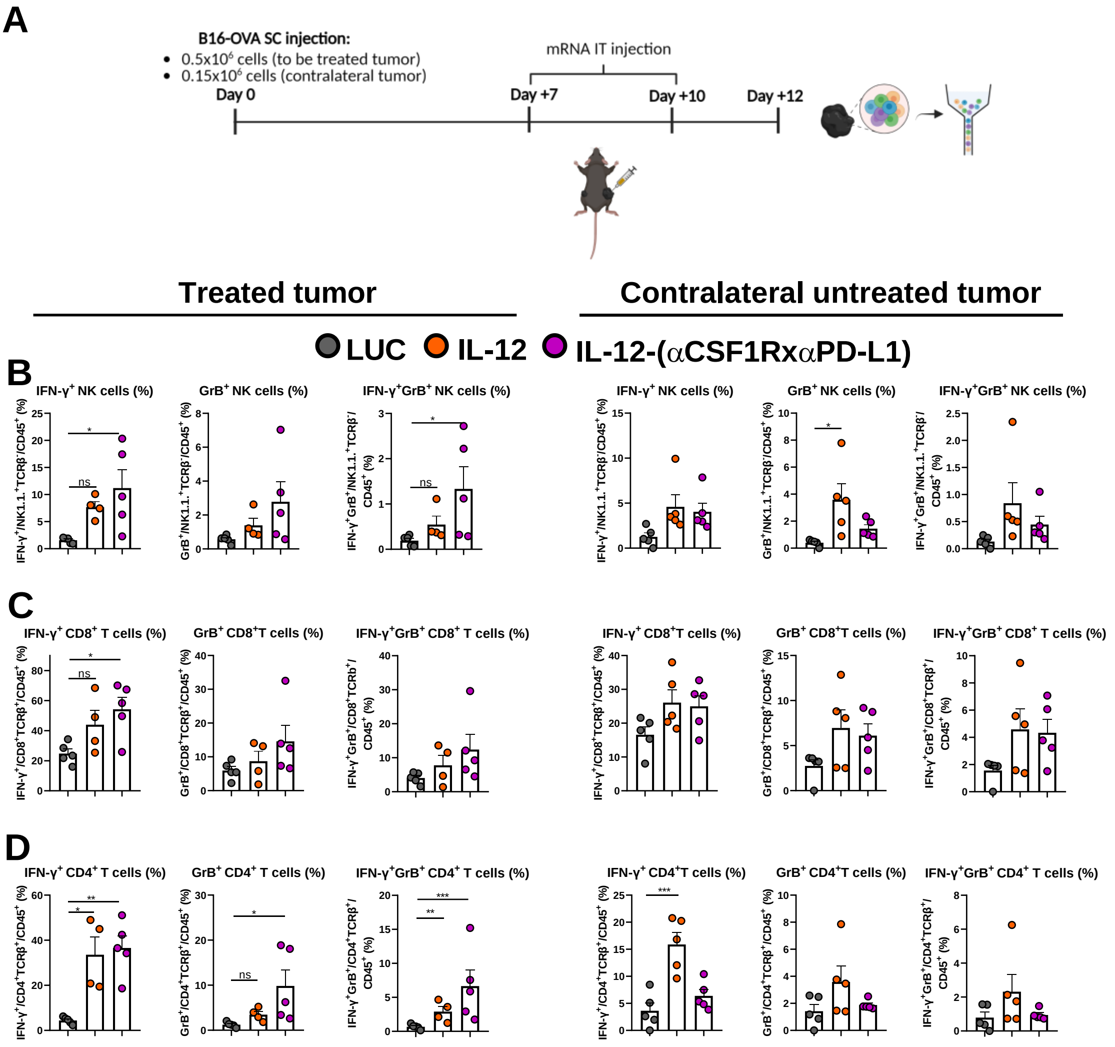

Figure S7. Expression of IFN-γ and granzyme B in T lymphocytes and NK cells infiltrating the treated and contralateral untreated tumors. A. Scheme summarizing the treatment regimen and the experimental timeline including the flow cytometry analysis. B16-OVA-bearing mice (n=5) received two doses of the indicated mRNAs on day 7 and day 10 after tumor injection and were sacrificed two days after the last treatment. 0.5 µg of IL-12-encoding mRNA and 10 µg of the mRNA encoding IL-12-(αCSF1RxαPD-L1) and LUC were used. Immune cells of the excised tumors were characterized by multi-color flow cytometry analysis. Data are shown as mean ± SEM. Statistical significance was

determined with one-way ANOVA followed by Tukey's multiple comparisons (* $p < 0.05$, ** $p < 0.01$).

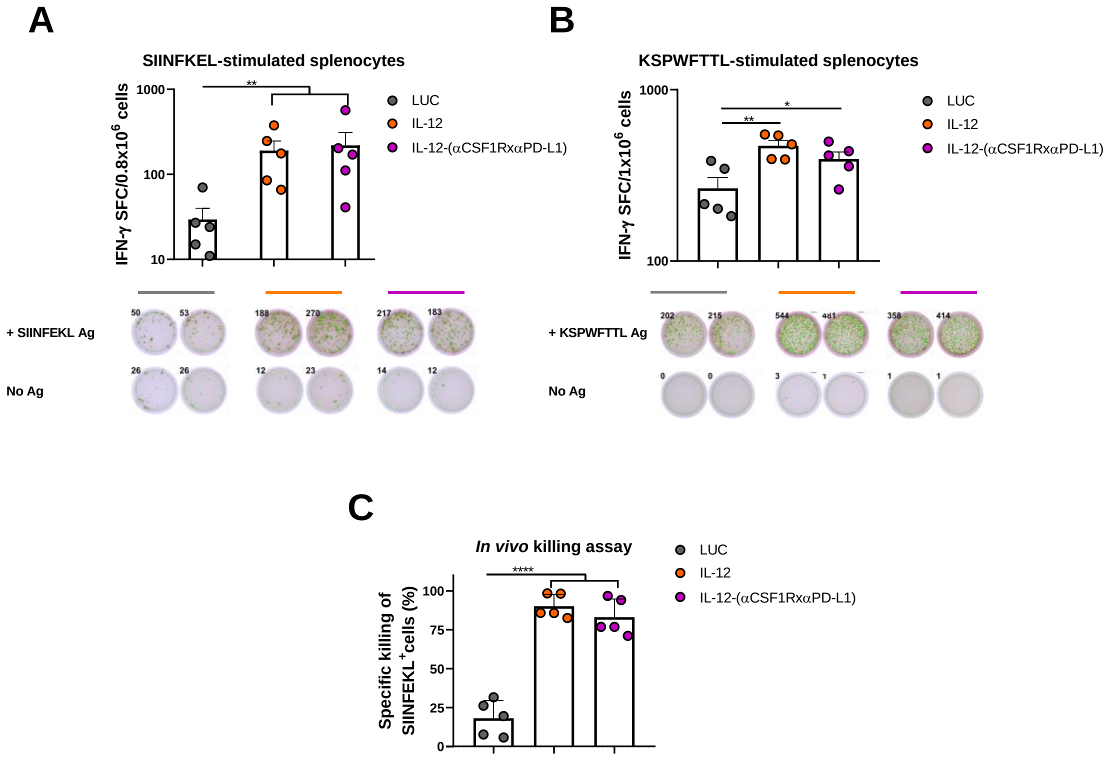

Figure S8. Targeted and untargeted IL-12 trigger a systemic antitumor immune response. A. C57BL/6 mice were injected with 0.5 x 10^6 B16-OVA cells subcutaneously in the right flank and 0.15 x 10^6 in the left flank (n=5/group). Mice received two injections of 10 µg of LUC-mRNA, 0.5 µg of IL-12-mRNA, 10 µg of IL-12-(αCSF1RxαPD-L1)-mRNA (on day 7 and 10 after tumor inoculation). Forty-eight hours after the second mRNA dose, 0.8 x 10^6 splenocytes were stimulated with 0.001 mg/ml $OVA_{257-264}$ peptide or with medium alone and incubated on an IFN-γ ELISpot plate. After 24 h, the spot-forming units were counted with an automated ImmunoSpot counter. Results correspond to data after subtraction of values obtained in control wells without peptide. Below the graph, two representative mice of each condition are shown. **B.** C57BL/6 mice were injected with 0.5 x 10^6 MC38 cells subcutaneously in the right flank and 0.2 x 10^6 in the left flank (n=5/group). Mice received two inoculations of 10 µg of LUC-mRNA, 0.5 µg of IL-12-mRNA, 10 µg of IL-12-(αCSF1RxαPD-L1)-mRNA (on day 6 and 9 after tumor inoculation). Forty-eight hours after the second mRNA dose, 1 x 10^6 splenocytes were

stimulated with 0.01 mg/ml KSPWFTTL peptide or with medium alone and incubated on an IFN-γ ELISpot plate. After 24 h, the spot-forming units were counted with an automated ImmunoSpot counter. Results correspond to data after subtraction of values obtained in control wells without peptide. Below each graph, two representative mice of each condition are shown. **C.** C57BL/6 mice were injected with 0.5 x 10^6 B16-OVA cells subcutaneously in the right flank and 0.15 x 10^6 in the left flank (n=5/group). Mice received three inoculations of 10 μg of LUC-mRNA, 0.5 μg of IL-12-mRNA, 10 μg of IL-12-(αCSF1RxαPD-L1)-mRNA (on day 7, 10 and 13 after tumor inoculation). In order to measure *in vivo* killing capability of $CD8^+$ T cells, single-cell suspensions of splenocytes were obtained from naive mice. Half of the splenocytes were pulsed for 30 mins at 37 ºC with 0.01 mg/ml $OVA_{257-264}$ peptide and the other half was incubated with the same volume of PBS only. After extensive washing, the two populations were stained with 5mM and 0.5mM of CFSE dye, respectively, and were mixed in a 1:1 ratio in order to retro-orbitally inject 10 x 10^6 cells per mouse 24 h after the third mRNA dose. 20 h post-transfer of the cells, spleens were isolated, processed and single-cell suspensions were analyzed by flow cytometry. To calculate the percentage of specific lysis of the target cell population, the following equation was used for each treated mouse, compared to naïve control: %specific lysis = 100 - [100*(%$CFSE^{high}$ immunized mouse/% $CFSE^{low}$ immunized mouse)/(% $CFSE^{high}$ naïve mouse/% $CFSE^{low}$ naïve mouse). Data are shown as mean ± SEM. Statistical significance was determined with one-way ANOVA followed by Dunnett's multiple comparison tests of Log-transformed data for panels A and B and followed by Tukey's multiple comparisons for panel C (*$p < 0.05$, **$p < 0.01$, ****$p < 0.0001$).

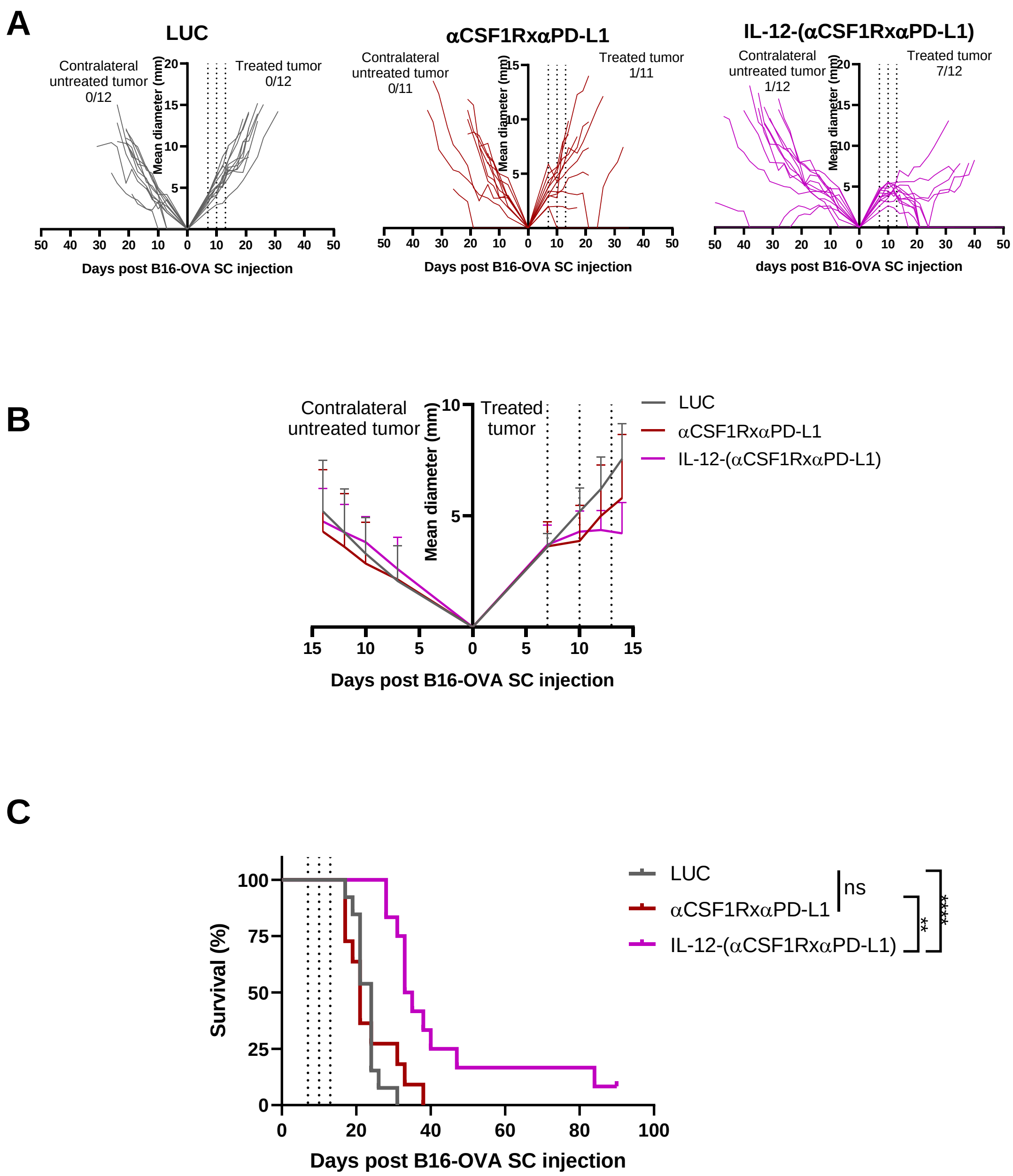

Figure S9. IL-12 is essential for the antitumor efficacy of the diabody-encoded mRNAs. Mice were injected with the B16-OVA cell line in both flanks and treated with the 10 µg of the indicated constructs on day 7, 10, and 13 after tumor cell inoculation. Two independent experiments were pooled (n=11-12). **A.** Individual follow-up of tumor sizes where the fraction of cured tumors is presented. **B.** Cumulative data of panel B. **C.** Mice survival follow-up of 2 pooled experiments is shown. Survival data in panel C were analyzed by Log-rank (Mantel-Cox) tests.

A

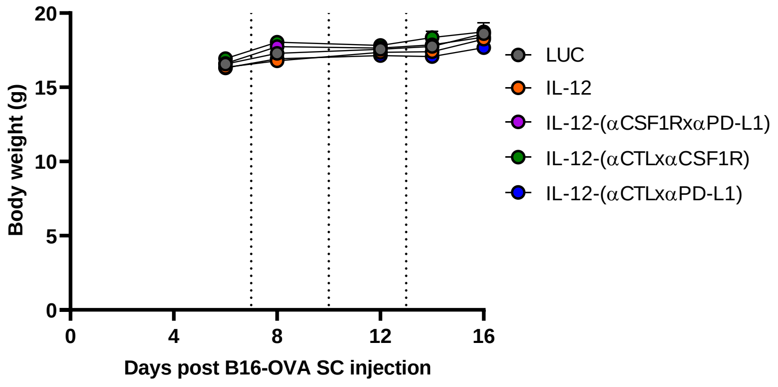

B

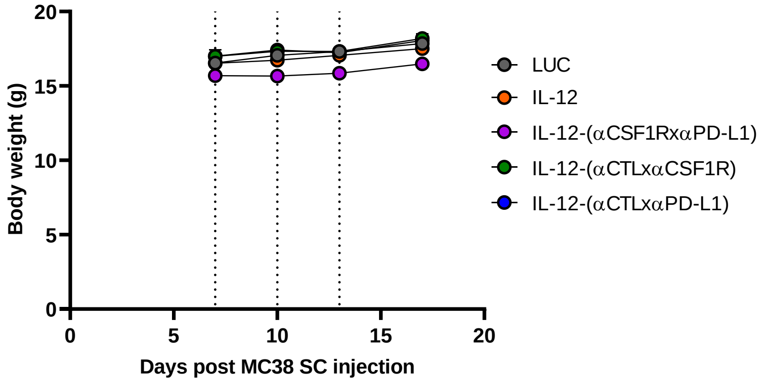

Figure S10. Treatments with mRNA-encoding IL-12-carrying constructs at the therapeutic dose do not induce toxicity in terms of weight loss. A. Mice of the antitumor experiments shown in Fig. 5B were weighted throughout the experiment starting from one day before the first mRNA injection. **B.** Mice of the antitumor experiments shown in Fig. 6B were weighed throughout the experiment, starting from just before the first mRNA injection. Statistical significance was determined with one-way ANOVA followed by Sidak's multiple comparisons tests. No significant differences were detected.

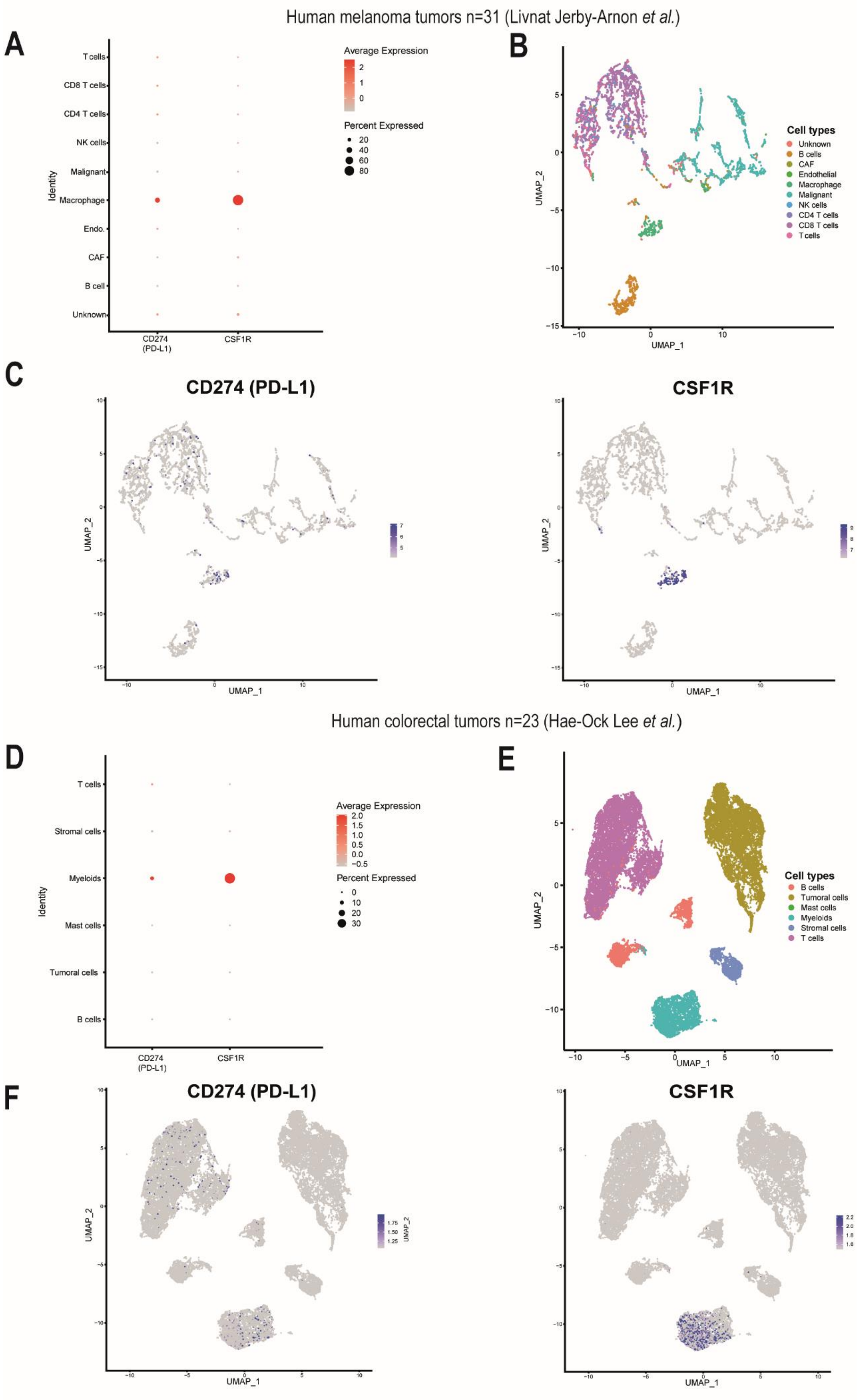
Human melanoma tumors n=31 (Livnat Jerby-Arnon *et al.*)
A
B
Average Expression
Percent Expressed
Identity
T cells
CD8 T cells
CD4 T cells
NK cells
Malignant
Macrophage
Endo.
CAF
B cell
Unknown
CD274 (PD-L1)
CSF1R
Cell types
Unknown
B cells
CAF
Endothelial
Macrophage
Malignant
NK cells
CD4 T cells
CD8 T cells
T cells
UMAP_1
UMAP_2
C
CD274 (PD-L1)
CSF1R
Human colorectal tumors n=23 (Hae-Ock Lee *et al.*)
D
E
Average Expression
Percent Expressed
Identity
T cells
Stromal cells
Myeloids
Mast cells
Tumoral cells
B cells
CD274 (PD-L1)
CSF1R
Cell types
B cells
Tumoral cells
Mast cells
Myeloids
Stromal cells
T cells
UMAP_1
UMAP_2
F
CD274 (PD-L1)
CSF1R

Figure S11. Evidence of expression of the transcripts of CSF1R and PD-L1 in the myeloid compartment of human tumor samples. Analysis of published scRNA-sequencing experiments of cohorts of 31 patients with melanoma tumors (**A-C**) and 23 patients with colorectal cancer (**D-F**). **A.** The dot plot represents the percentage of each indicated cell type expressing CD274 (PD-L1) and CSF1R transcripts and the intensity of such expression. **B.** The UMAP represents a dimension-reduced projection of different color-coded cell types. **C.** The UMAPs highlight the intensity of expression of the targets of interest by the different cell types. **D-F.** As described in A-C.
