# Supplementary material for: Intratumoral injection of IL-12-encoding mRNA targeted to CSFR1 and PD-L1 exerts potent anti-tumor effects without substantial systemic exposure
Source: Mol Ther Nucleic Acids. 2023 Jul 19;33:599–616. doi: 10.1016/j.omtn.2023.07.020 (PMC10450355; doi:10.1016/j.omtn.2023.07.020)
Supplement: Document S2. Article plus supplemental information [file mmc2.pdf]

Molecular Therapy
Nucleic Acids
Original Article

Intratumoral injection of IL-12-encoding mRNA targeted to CSFR1 and PD-L1 exerts potent anti-tumor effects without substantial systemic exposure

Claudia Augusta Di Trani,[1,2] Assunta Cirella,[1,2] Leire Arrizabalaga,[1,2] Maite Alvarez,[1,2,3] Ángela Bella,[1,2] Myriam Fernandez-Sendin,[1,2] Joan Salvador Russo-Cabrera,[1,2] Celia Gomar,[1,2] Nuria Ardaiz,[1,2] Alvaro Teijeira,[1,2,3] Elixabet Bolaños,[1,2] José González-Gomariz,[1,2] Itziar Otano,[1,2] Fernando Aranda,[1,2] Belén Palencia,[1] Aina Segués,[4,5] Shuyu Huang,[4,5] Sander M.J. van Duijnhoven,[6] Andrea van Elsas,[7] Ignacio Melero,[1,2,3,8,9,10] and Pedro Berraondo[1,2,3,10]

[1]Program of Immunology and Immunotherapy, Cima Universidad de Navarra, Pamplona, Spain; [2]Navarra Institute for Health Research (IDISNA), Pamplona, Spain; [3]Spanish Center for Biomedical Research Network in Oncology (CIBERONC), Madrid, Spain; [4]Institute of Immunology and Infection Research, School of Biological Sciences, University of Edinburgh, EH9 3FL Edinburgh, UK; [5]Faculty of Veterinary Medicine, Department of Infectious Diseases and Immunology, Utrecht University, 3584 CS Utrecht, The Netherlands; [6]ImmunoPrecise Antibodies Ltd., 5349 AB Oss, The Netherlands; [7]Third Rock Ventures, San Francisco, CA 94158, USA; [8]Department of Immunology and Immunotherapy, Clínica Universidad de Navarra, Pamplona, Spain; [9]Department of Oncology, Clínica Universidad de Navarra, Pamplona, Spain

IL-12 is a potent cytokine for cancer immunotherapy. However, its systemic delivery as a recombinant protein has shown unacceptable toxicity in the clinic. Currently, the intratumoral injection of IL-12-encoding mRNA or DNA to avoid such side effects is being evaluated in clinical trials. In this study, we aimed to improve this strategy by further favoring IL-12 tethering to the tumor. We generated *in vitro* transcribed mRNAs encoding murine single-chain IL-12 fused to diabodies binding to CSF1R and/or PD-L1. These targeted molecules are expressed in the tumor microenvironment, especially on myeloid cells. The binding capacity of chimeric constructs and the bioactivity of IL-12 were demonstrated *in vitro* and *in vivo*. Doses as low as 0.5 μg IL-12-encoding mRNA achieved potent antitumor effects in subcutaneously injected B16-OVA and MC38 tumors. Treatment delivery was associated with increases in IL-12p70 and IFN-γ levels in circulation. Fusion of IL-12 to the diabodies exerted comparable efficacy against bilateral tumor models. However, it achieved tethering to myeloid cells infiltrating the tumor, resulting in nearly undetectable systemic levels of IL-12 and IFN-γ. Overall, tethering IL-12 to intratumoral myeloid cells in the mRNA-transferred tumors achieves similar efficacy while reducing the dangerous systemic bioavailability of IL-12.

INTRODUCTION

The clinical approval of recombinant interleukin-2 (IL-2) and interferon-α (IFN-α) cytokines for treating several malignant diseases marked a milestone in cancer immunotherapy, even if based on rather limited efficacy. Indeed, these early immunotherapies achieved only modest therapeutic effects and were associated with severe immune-related adverse events.[1] In recent years, cancer immunotherapy has experienced rapid progress because of the success of chimeric antigen receptor (CAR)-T cell therapy against hematological malignancies,[2] and to the unprecedented efficacy of immune checkpoint inhibitors against multiple solid tumors.[3] Of note, to date, monoclonal antibodies (mAbs) targeting the programmed cell death-1 (PD-1)/programmed death ligand 1 (PD-L1) pathway have been approved for 23 different indications.[4] Nonetheless, the increased overall survival rate has so far been limited to a fraction of treated patients, and in many tumor types, there is only a marginal benefit. As a consequence, preclinical and clinical research is needed to combat resistance to immunotherapy.[5] Combined immunotherapy is one of the routes to increase efficacy. These efforts have led to the first approvals of the combinations of anti-PD-1/PD-L1 mAbs with chemotherapy, antiangiogenic drugs, or other immuno-oncology agents such as anti-CTLA-4 (cytotoxic T lymphocyte antigen-4) and anti-LAG-3 (lymphocyte-activation gene-3) mAbs.[6] One of the most potent anticancer immunotherapy weapons is IL-12, a heterodimeric cytokine encompassing the two independently produced p40 and p35 subunits linked by disulfide bonds. IL-12 is secreted mostly by activated conventional type 1 dendritic (cDC1) antigen-presenting cells and interacts with its heterodimeric receptor expressed on natural killer (NK), NKT, and activated T cells.[7] IL-12 signals mainly via JAK-STAT-4.[8] Chiefly, such immune cells will secrete IFN-γ.[9] In the cancer context, the IL-12-IFN-γ axis strongly promotes modulation

Received 8 March 2023; accepted 17 July 2023;
https://doi.org/10.1016/j.omtn.2023.07.020.

[10]Senior author

Correspondence: Pedro Berraondo, Cima Universidad de Navarra, Avenida Pio XII 55, 31008 Pamplona, Spain.
E-mail: pberraondol@unav.es

This is an open access article under the CC BY license (http://creativecommons.org/licenses/by/4.0/).

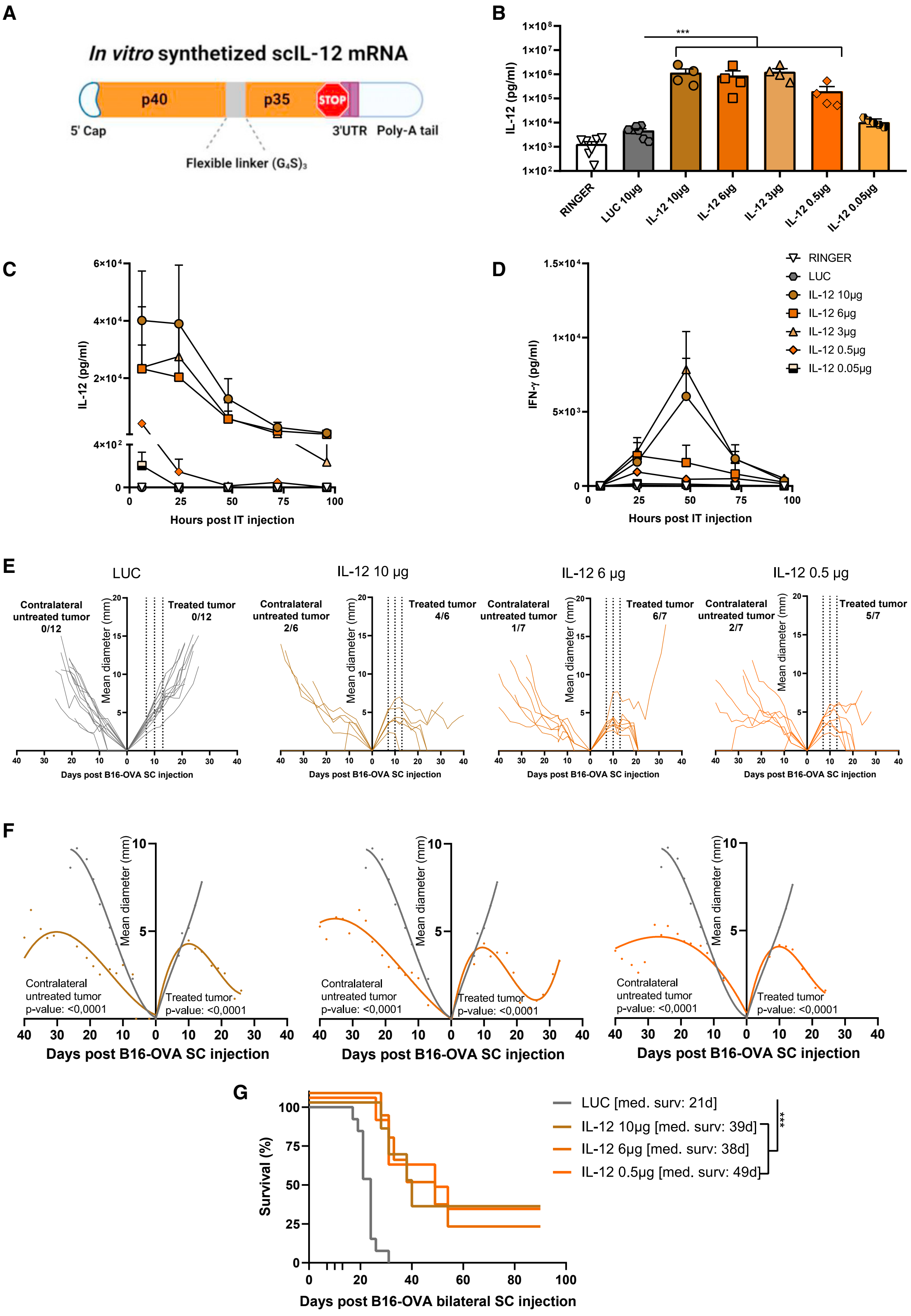

(legend on next page)

of the inflammatory infiltrates in the tumor microenvironment. Indeed, IFN-γ is responsible for neo-angiogenesis inhibition,[10] reprogramming of tumor-associated macrophages,[11] enhanced tumor antigen presentation,[12] and leukocyte recruitment.[13] Of note, the IL-12-IFN-γ axis further amplifies IL-12 secretion in a positive regulatory loop.[7] Nevertheless, this powerful antitumor activity is accompanied by severe systemic side effects that limit the application of IL-12-based therapy in the clinic when used as a recombinant protein.[14,15] Many techniques are currently being exploited at a preclinical and clinical level to avoid toxicity dependent on systemic sustained IL-12 expression.[16] One example is the use of messenger RNA (mRNA)-based technology (NCT03946800),[17] which takes advantage of the low intrinsic half-life of mRNA molecules. Short local expression limits the potential side effects derived from sustained expression. Local and safer IL-12 bioavailability opens up opportunities for multiple injection regimens and for the mixture of synergistic cytokines (NCT03871348).[18–20] Furthermore, mRNA-based technology overcomes many of the drawbacks of the recombinant protein-based technology, such as production, purification, and post-translational modification issues, hence conceivably resulting in a more cost-effective platform.[21] Direct intratumoral delivery is another strategy used to maximize the therapeutic index of the immunomodulatory molecule, thus favoring *in situ* bioavailability while limiting the on-target off-tumor toxicity of the injected molecule. Many aspects of the applicability of intratumoral delivery must be improved, but several clinical trials are currently evaluating local delivery and targeting strategies.[22,23]

Additional methods that have been used to avoid systemic leakage of IL-12 include its fusion to collagen-binding domains, to a masking peptide selectively cleaved in the tumor microenvironment, or to tumor-targeting antibodies.[16,24] For instance, NHS-IL-12 is an immunocytokine targeting the necrotic tumor areas expressing double-stranded DNA, which is at present evaluated in a phase II clinical trial (NCT04633252).[25]

We previously showed that upon intratumoral injection in Ringer's lactate solution, naked mRNA is correctly intratumorally translated into a polypeptide containing IL-12.[20] Here we express mRNAs encompassing three components: (1) single-chain IL-12 (scIL-12), which consists of a single open reading frame containing the p40 and p35 subunits fused by a flexible linker[26]; (2) the variable regions of the rat anti-mouse AFS98 mAb that binds colony-stimulating factor 1 receptor (CSF1R); (3) and the variable regions of avelumab, a human mAb that specifically binds to the human and mouse PD-L1 protein. When using these mRNA constructs given intratumorally, we observed antitumor efficacy mainly attributable to scIL-12, but fusion to the diabodies conferred tethering features to the cytokine, such that it becomes retained by myeloid cells in the tumor microenvironment. Confinement to the site where IL-12 is expressed prevents dangerous systemic exposure to IL-12.

RESULTS

Intratumoral injection of naked scIL-12 mRNA is correctly expressed *in vivo* and increases overall survival in bilateral B16-OVA tumor-bearing mice

To assess the antitumor activity of immunostimulatory molecules expressed in the tumor microenvironment, we took advantage of the capacity of Ringer's lactate solution to allow expression of the proteins encoded by naked mRNA upon intratumoral injection.[19,20,27–29] We first evaluated this delivery method by injecting 10 μg of a luciferase (LUC)-encoding mRNA formulated in 50 μL of Ringer's lactate solution into subcutaneously implanted B16-OVA tumors. The bioluminescence catalyzed by the *in vivo* translated reporter protein was measured over time, as summarized in Figure S1A. LUC mRNA-injected mice showed a significantly higher photon emission level compared with mice injected with vehicle 6 h after mRNA administration (Figure S1B). Then the measurable LUC activity slowly decreased with a half-life of 10.5 h (Figure S1C).

Next, in order to *in vitro* characterize the scIL-12-encoding mRNA (schematized in Figure 1A), we transfected B16-OVA cell line with IL-12 mRNA or a GFP mRNA formulated with the TransIT-mRNA transfection kit. As shown in Figure S2A, IL-12 was correctly translated and secreted, as measured in the supernatants collected 72 h after transfection using ELISA. To assess the bioactivity of the encoded molecule in terms of its capability to trigger phosphorylation of Y693 of STAT-4 and IFN-γ production, we incubated pre-activated splenocytes with the conditioned media containing the translated IL-12 or with recombinant IL-12 at the same concentration. IL-12 from both sources significantly increased the phosphorylation level of STAT-4 and IFN-γ production to a similar degree (Figures S2B and S2C). Next, we sought to investigate the *in vivo* translation and secretion efficiency of the scIL-12-encoding mRNA following the intratumoral delivery method in Ringer's lactate solution. To this end, we administered different doses of the mRNA

Figure 1. Intratumoral injection of naked single-chain IL-12 mRNA increases overall survival in bilateral B16-OVA implanted mice

(A) Image schematizing the sequence of the *in vitro* transcribed mRNA encoding scIL-12. (B–D) C57BL/6 mice (n = 4–7) were injected with 0.5×10^6 B16-OVA cells subcutaneously in the right flank. Seven days post-tumor inoculation, the indicated doses of mRNAs were delivered intratumorally in 50 μL of Ringer's lactate solution. (B) Six hours post-mRNA injection, tumors were excised and homogenized in the presence of a protease inhibitor cocktail. IL-12p70 levels were determined using ELISA. (C and D) At the indicated time points, sera of mice were collected, and ELISA was performed to detect IL-12 and IFN-γ. (E–G) C57BL/6 mice were injected with 0.5×10^6 B16-OVA cells subcutaneously in the right flank and 0.15×10^6 in the left flank. Luciferase-encoding mRNA (10 μg) and the indicated doses of IL-12-encoding mRNAs were injected into the right tumor on days 7, 10, and 13 (as indicated by the dashed lines). (E) Individual follow-up of tumor sizes with the fraction of cured tumors is presented. (F) Compiled data and statistical comparisons of results shown in (E). (G) Survival follow-up of two independent pooled experiments is shown (LUC, n = 12; IL-12 10 μg, n = 6; IL-12 6 μg, n = 7; IL-12 0.5 μg, n = 7). Data are expressed as mean ± SEM. Statistical significance was determined using one-way ANOVA followed by Dunnett's multiple-comparison tests of log-transformed data in (B). In (F), longitudinal data were fitted to a third-order polynomial equation and compared using an extra sum-of-squares F test. Survival data in (G) were analyzed using log rank (Mantel-Cox) tests (***$p < 0.001$).

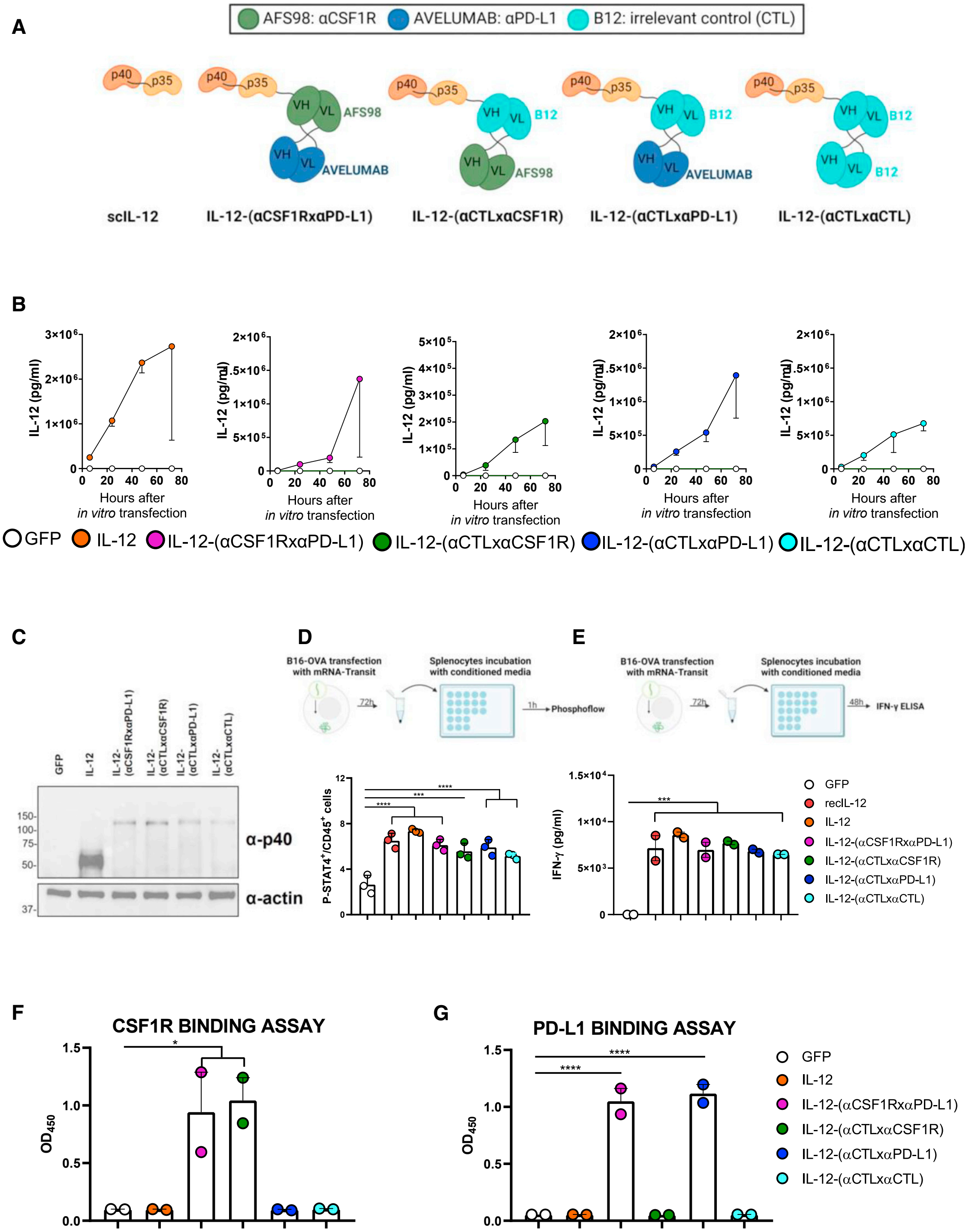

Figure 2. IL-12-fused diabodies retain the activity of each moiety

(A) Schematic representation of the different mRNA-encoded constructs. (B) B16-OVA cells were transfected with each of the indicated mRNA formulated with the TransIT-mRNA Transfection kit. Supernatants were collected 6, 24, 48, and 72 h post-transfection and assayed for IL-12p70 detection using ELISA. (C) Twenty-four hours after transfection, cells were lysed, proteins extracted, and a western blot was performed on a 10% SDS-PAGE in denaturing conditions with antibodies against IL-12-p40 subunit and α-actin. (D) Murine splenocytes were pre-activated O/N using plate-bound anti-CD3 and soluble anti-CD28. The day after, 3×10^6 pre-activated splenocytes were incubated for 1 h at 37°C with the conditioned media from mRNA-transfected B16-OVA cells, containing 400 ng IL-12, as previously assessed by the IL-12p70 ELISA

(legend continued on next page)

and, 6 h post-injection, we analyzed using ELISA IL-12p70 concentrations in the recovered B16-OVA tumor homogenates (Figure 1B) or in the sera of mice over time (Figure 1C). Compared with mice injected with vehicle alone or LUC-mRNA, the administration of the IL-12 mRNA at doses ranging from 10 to 0.5 μg resulted in significantly higher levels of IL-12p70 in the injected tumor nodule (Figure 1B). Six hours after delivery, systemic levels of IL-12 were also detectable at doses ranging from 10 to 0.5 μg. When the highest doses of 10, 6, and 3 μg were used, IL-12p70 serum levels were detected up to 48 h post-injection, whereas the expression after injection of 0.5 μg was only detected up to 24 h post-injection (Figure 1C). The main downstream effector of IL-12-mediated antitumor activity is IFN-γ,[30,31] and we used the concentrations of this cytokine in the sera of the mice as a readout of IL-12 bioactivity. In the case of mice injected with 10 and 3 μg, IFN-γ levels peaked at 48 h post-mRNA delivery, with 5 of 6 mice still showing measurable levels up to 72 h in both cases. However, in the case of mice injected with 6 and 0.5 μg of mRNA, the peak was reached one day after injection, and the concentration decreased over time, with only a few mice presenting detectable levels 72 h after the injection (Figure 1D). Hence, even when low doses of IL-12 mRNA were used, systemic IL-12 and IFN-γ could be detected. Of note, IFN-γ is reported to be the main downstream mediator of IL-12 toxicity.[14,15,32]

To ascertain the therapeutic potential, we comparatively evaluated the antitumor effects of different doses of IL-12-encoding mRNA in a bilateral subcutaneously implanted B16-OVA tumor model. The tumor implanted in the right flank was treated with three doses of the therapeutic or control mRNA every three days. The potential abscopal effect triggered by the local administration of the mRNA was also evaluated in the distant tumor inoculated in the left flank that remained uninjected. As shown in Figures 1E and 1F, the delivery of irrelevant control mRNA did not result in the rejection of any of the primary or untreated tumors. In contrast, the three evaluated mRNA doses exerted a potent antitumor effect in the treated lesion with total disappearance of the tumors in at least 50% of mice. Additionally, the treatment limited the growth of the distant untreated lesions, achieving tumor rejections, albeit in a few cases. As a consequence, the three different IL-12 mRNA doses significantly increased overall survival compared with the negative control group. Interestingly, no statistical differences were found among the three tested doses of IL-12 mRNA (Figure 1G). Importantly, a three-dose regimen attained better antitumor efficacy and significantly higher overall survival than one or two mRNA injections (Figures S3A and S3B).

All in all, the intratumoral delivery of naked scIL-12 in Ringer's lactate solution results in detectable levels of IL-12p70 both in the tumor niche and in circulation, and it can attain antitumor efficacy, even when as little as 0.5 μg of the therapeutic mRNA are injected.

Functionality of mRNA-encoded immunocytokines encompassing IL-12, anti-CSF1R and/or anti-PD-L1

Systemic IL-12 exposure may lead to unwanted side effects such as those that have been observed in human clinical trials.[14,15] Given that even the lowest therapeutic dose of the IL-12-encoding mRNA still resulted in the detectable levels of IL-12 and IFN-γ in the periphery (Figures 1C and 1D), we produced mRNAs encoding IL-12-fused diabodies designed to tether the cytokine to the tumor milieu. As binding moieties, we selected the variable regions of an antibody reactive to murine CSF1R (clone AFS98) and a cross-reactive antibody recognizing human and mouse PD-L1 (clone avelumab). As an irrelevant binding negative control (CTL), we used the variable regions of an antibody targeting a glycoprotein of the human immunodeficiency virus type 1 (HIV-1) envelope (B12 clone). We designed different single-chain mRNA sequences to encode several combinations of the three moieties fused by flexible linkers: the bispecific IL-12-(αCSF1RxαPD-L1), the CSF1R-targeting IL-12-(αCTLxαCSF1R), the PD-L1-targeting IL-12-(αCTLxαPD-L1), and the irrelevant immunocytokine IL-12-(αCTLxαCTL), as represented in Figure 2A.

To characterize such trimeric molecules *in vitro*, we transfected B16-OVA cell lines with the different mRNAs formulated with the TransIT-mRNA transfection kit. IL-12p70 levels were measured using ELISA in the supernatants of cells collected over time. Starting from 24 h after the transfection, increasing levels of IL-12p70 were recovered in the supernatants of cells transfected with all the mRNA constructs. In contrast, no IL-12 was measured in the supernatants from cells transfected with a GFP-encoding mRNA (Figure 2B). Of note, a different efficiency of translation was observed between IL-12 and IL-12 diabody-encoded chimeras. To assess that the molecular weight of the fusion proteins corresponded to the expected size, 24 h after mRNA transfection, cells were lysed, and the protein pool of each experimental condition was run on SDS-PAGE. The separated proteins were transferred to a nitrocellulose membrane, and immunoblotting with an anti-IL-12p40 mAb confirmed the expected molecular weights of each construct (Figure 2C). In order to evaluate the bioactivity of the scIL-12 fused to the heterodimeric diabodies, murine splenocytes were pre-activated overnight (O/N), and then incubated with chimera-enriched

sandwich assay. As a positive control, 400 ng recombinant IL-12 were used, and the supernatant of cells transfected with GFP-mRNA was used as a negative control. Then, cells were washed and the pellet was resuspended in Cytofix buffer for 15 min at 37°C. After extensive washing, cells were permeabilized using Perm Buffer III for 30 min at 4°C and then stained with anti-CD45-PerCP and anti-p-STAT-4-PE antibody for 45 min at RT. The percentage of $CD45^+$ cells with phosphorylated STAT-4 are shown. (E) Murine splenocytes were pre-activated in an anti-CD3-coated plate O/N. Then, they were treated with supernatants collected 72 h after mRNA transfection of B16-OVA cells or with 50 ng recombinant IL-12 as a positive control. Forty-eight hours after incubation, the splenocyte culture supernatants were analyzed for IFN-γ production using ELISA. (F and G) Nunc-maxisorp plates were coated with CSF1R (F) or PD-L1 (G) recombinant protein and incubated with supernatants collected 72 h after mRNA transfection of B16-OVA cells. The binding to the targets was detected using an HRP-fused-anti-IL-12 antibody. Data are shown as mean ± SEM. Statistical significance was determined using one-way ANOVA with Dunnett's multiple-comparisons test (*p < 0.05, ***p < 0.001, and ****p < 0.0001).

supernatants for 48 h. Phosphorylation of Y693 of STAT-4 and IFN-γ secretion by such pre-activated splenocytes were used as a readout for IL-12 bioactivity. As shown in Figures 2D and 2E, all IL-12-containing constructs induced the phosphorylation of STAT-4 and release of IFN-γ. No significant differences in IL-12 bioactivity were observed among the different constructs and with respect to recombinant IL-12. Finally, to evaluate the binding capacity of each moiety of the diabody, we performed two binding assays. ELISA plates were coated with CSF1R or PD-L1 recombinant proteins and incubated with culture supernatants of cells transfected with the different mRNAs. The targeting capacity was qualitatively ascertained by detecting the bound construct through an anti-IL-12-HRP (horseradish peroxidase) antibody. The IL-12-bispecific diabody retained the ability to bind both targets, while the IL-12 fused to the anti-CSF1R-targeting arm or the IL-12 fused to the anti-PD-L1-targeting moiety bound only to their respective targets. Finally, the IL-12 fused to the irrelevant binding arms did not bind to any of the recombinant proteins (Figures 2F and 2G).

In order to study if the chimeric constructs were able to bind to myeloid cells infiltrating the tumors, we devised an *ex vivo* tethering experiment. For this purpose, B16-OVA tumors excised from mice 11 days after subcutaneous inoculation were used to generate single-cell suspensions and were incubated with culture supernatants derived from B16-OVA cells transfected with the different mRNAs, containing 460 ng IL-12 in all conditions, as previously assessed by a p70/IL-12 ELISA sandwich assay. Subsequently, cells were washed and co-stained with a cocktail of fluorochrome-labeled antibodies to identify which myeloid populations were decorated with IL-12 on their surface. This experiment permitted a readout of diabodies' capability to tether the IL-12 cytokine to the tumor immune microenvironment (Figure 3A). As shown in Figure 3B, compared with cells incubated with IL-12-enriched supernatants, a significantly higher percentage of macrophages and monocytic myeloid-derived suppressor cells (mo-MDSC) cells displayed IL-12 on their surface if incubated with IL-12 fused to the binding diabodies. Of note, IL-12-(αCSF1RxαPD-L1) and IL-12-(αCTLxαPD-L1) showed a higher tethering capacity than IL-12-(αCTLxαCSF1R). These two diabodies in the chimeric proteins were also able to significantly tether IL-12 to the surface of the polymorphonuclear (PMN)-MDSC population compared with unfused IL-12. The myeloid-cell gating strategy used in this experiment is shown in Figure S4A. From these results, we conclude that our *in vitro* transcribed single-chain mRNA molecules are successfully translated and secreted by murine tumor cells into fusion polypeptides in which each module retains its activity *in vitro* and *ex vivo*.

mRNA-encoded IL-12 diabodies are functional *in vivo* and exert local and systemic antitumor effects in bilateral tumor models

To investigate if IL-12 fused to the diabodies also retained its bioactivity *in vivo*, we first evaluated the mouse signs of toxicity after administering 10 μg of the mRNA encoding IL-12 or IL-12-(αCSF1RxαPD-L1) on day 7, 10, and 13 after tumor cell inoculation. At the end of the treatment, mice treated with IL-12 showed a reduced body weight, high levels of circulating IL-12 and IFN-γ, splenomegaly, and elevated transaminases. Interestingly, these adverse effects were absent in mice treated with the IL-12 fused to the diabody (Figures S5A–S5F). To further characterize the *in vivo* activity of the different constructions, we used multi-color flow cytometry analysis of single-cell suspensions derived from excised tumors four days after the second mRNA dose that were stained with fluorochrome-labeled antibodies (Figure 4A). For each construction, the mRNA dose for *in vivo* delivery was selected on the basis of its *in vivo* translation efficiency, to attain similar levels of expression of IL-12p70 in the tumor microenvironment. As shown in Figure 4B, mice treated with IL-12-(αCTLxαPD-L1) presented a significantly lower percentage of the macrophage population compared with LUC-injected mice. Compared with mice injected with LUC-mRNA, all IL-12-carrying constructs induced a decrease in cells expressing the M2-like marker CD206 and increased those with the M1-like markers MHC-II^{hi}CD38 on their surfaces, among the macrophage population. Moreover, we observed a significant increase in the percentage of mo-MDSC in the IL-12-(αCSF1RxαPD-L1) diabody compared with the LUC-mRNA condition. In contrast, the IL-12-(αCTLxαPD-L1) diabody increased the PMN-MDSC subset. Even though we did not find a significant change in the percentage of CD8$^+$ T cells, we found an up-regulation in T cells of the activation marker CD25 and the proliferation marker Ki-67 in those mice treated with all the IL-12-bearing constructs, compared with those in the negative control group (Figure 4C). Additionally, IL-12-(αCTLxαPD-L1) treatment promoted a decrease of the exhaustion markers PD-1$^+$/Tim-3$^+$ on the surface of CD8$^+$ T lymphocytes (Figure 4C). As in the case of CD8$^+$ T cells, NK cells showed a more proliferative phenotype in tumors treated with all the IL-12-bearing constructs (Figure 4D). Furthermore, the IL-12-based treatment induced CD4$^+$ T cell proliferation. Interestingly, treatments with the IL-12-monospecific diabodies significantly decreased the percentage of regulatory T cells in the tumors (Figure 4E). The IL-12-(αCSF1RxαPD-L1) diabody resulted in a significantly higher infiltration of CD3$^+$, CD8$^+$ T cells, and NK cells, compared with LUC and untethered IL-12 constructs. Moreover, both IL-12-(αCSF1RxαPD-L1) and IL-12-(αCTLxαCSF1R) resulted in increased numbers of tumor-specific T cells in the treated tumor (Figure S6A). The only difference detected in tumor-infiltrating lymphocytes with respect to negative controls in the uninjected lesion was an increase in the proliferation of CD8$^+$ T cells in those mice treated with IL-12, IL-12-(αCTLxαCSF1R), and IL-12-(αCTLxαPD-L1) (Figures S6B and S6C). The corresponding fluorescence-activated cell sorting (FACS) gating strategy is shown in Figures S4A and S4B. To further characterize the functional status of the tumor-infiltrating T cells, we evaluated IFN-γ and granzyme B (GrB) in NK cells, CD4$^+$ and CD8$^+$ T lymphocytes in bilateral B16-OVA-bearing mice treated with mRNAs encoding LUC, IL-12, and IL-12-(αCSF1RxαPD-L1) injected in the tumor implanted in the right flank (Figure S7A). IL-12-(αCSF1RxαPD-L1) treatment significantly increased the percentage of IFN-γ$^+$GrB$^+$ NK cells and IFN-γ$^+$CD8$^+$ T cells in the injected lesion (Figures S7B and S7C, left panels). The treatment with untethered IL-12 resulted in a slight increase of these populations, compared with LUC condition, although not reaching statistical significance. Strikingly, both IL-12-based treatments significantly raised the percentage of

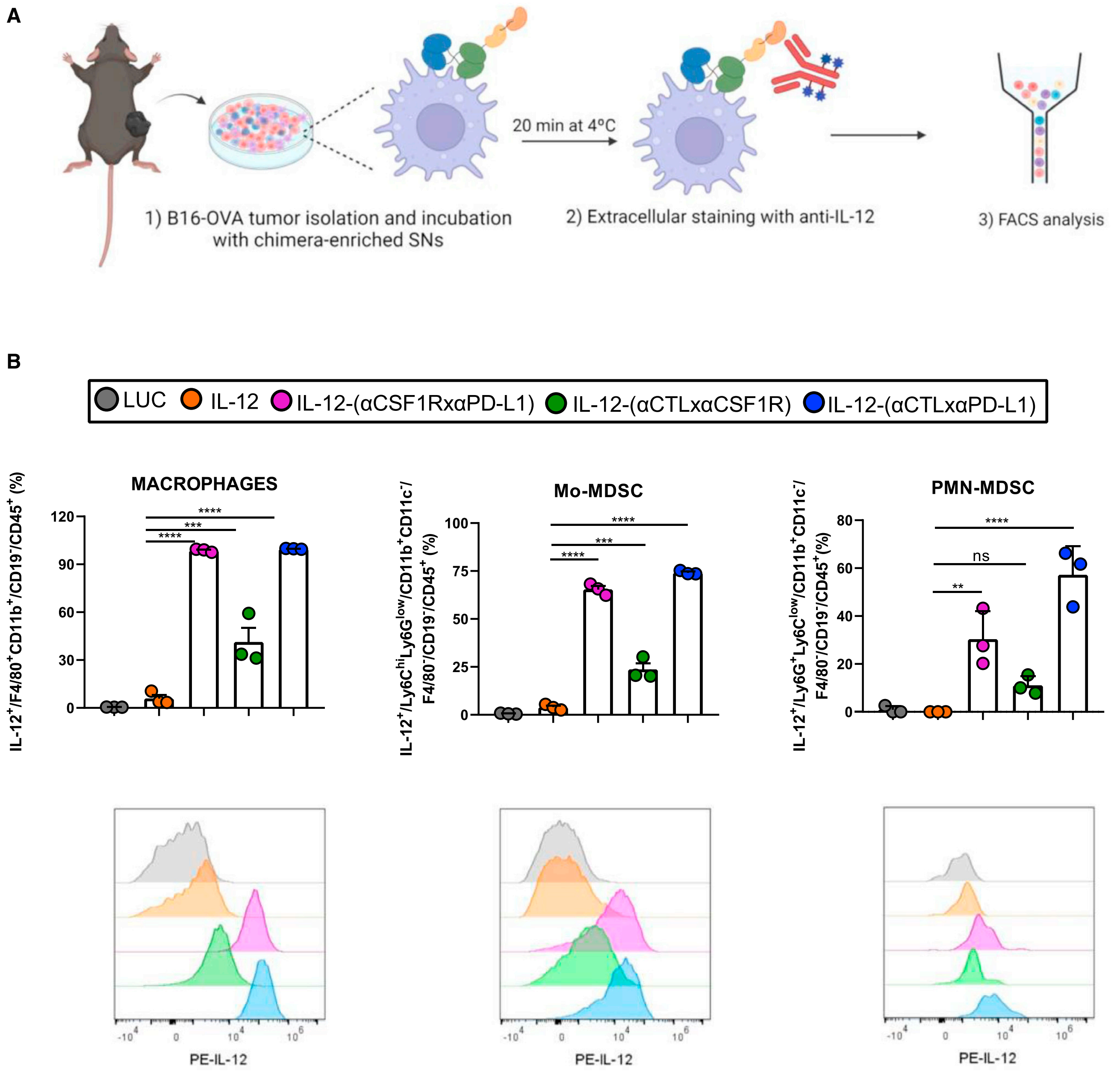

Figure 3. Evidence of the *ex vivo* tethering ability of IL-12 diabodies to myeloid populations in the tumor microenvironment (TME)
(A) Scheme of the experiment presented in (B). B16-OVA tumors were excised 11 days post-injection, processed, and *ex vivo* incubated with supernatants derived from B16-OVA cells transfected with the different chimeric constructs containing the same concentrations of IL-12 for all conditions (n = 3/condition). Subsequently, cells were washed and stained with a cocktail of fluorochrome-labeled antibodies to identify which myeloid populations presented IL-12 on their surface. (B) The percentage of macrophages, mo-MDSC, and PMN-MDSC displaying IL-12 on their surface is shown. Macrophages were gated as viable $CD45^+CD19^-CD11b^+F4/80^+$. The mo-MDSC population was classified as viable $CD45^+CD19^-F4/80^-CD11c^-CD11b^+$ $LY6C^{high}LY6G^{low}$ and the PMN-MDSC as viable $CD45^+CD19^-F4/80^-CD11c^-CD11b^+$ $LY6C^{low}LY6G^+$. A histogram overlay shows the flow cytometry analysis of IL-12 surface detection of one representative sample of each condition for each population. Data are expressed as mean ± SEM, and statistical significance was determined using one-way ANOVA followed by Dunnett's multiple-comparison tests in (B) (**p < 0.01, ***p < 0.001, and ****p < 0.0001).

IFN-γ^+GrB$^+$CD4$^+$ T cells, and the tethered IL-12 did so more pronouncedly (Figure S7D, left panel). As for the contralateral tumor (Figures S7B and S7D, right panel), scIL-12 induced a significant increase of GrB staining in NK cells and IFN-γ among CD4$^+$ T cells, which was not observed in the case of the trimeric molecule. Both IL-12 constructs showed a tendency to increase the percentage of

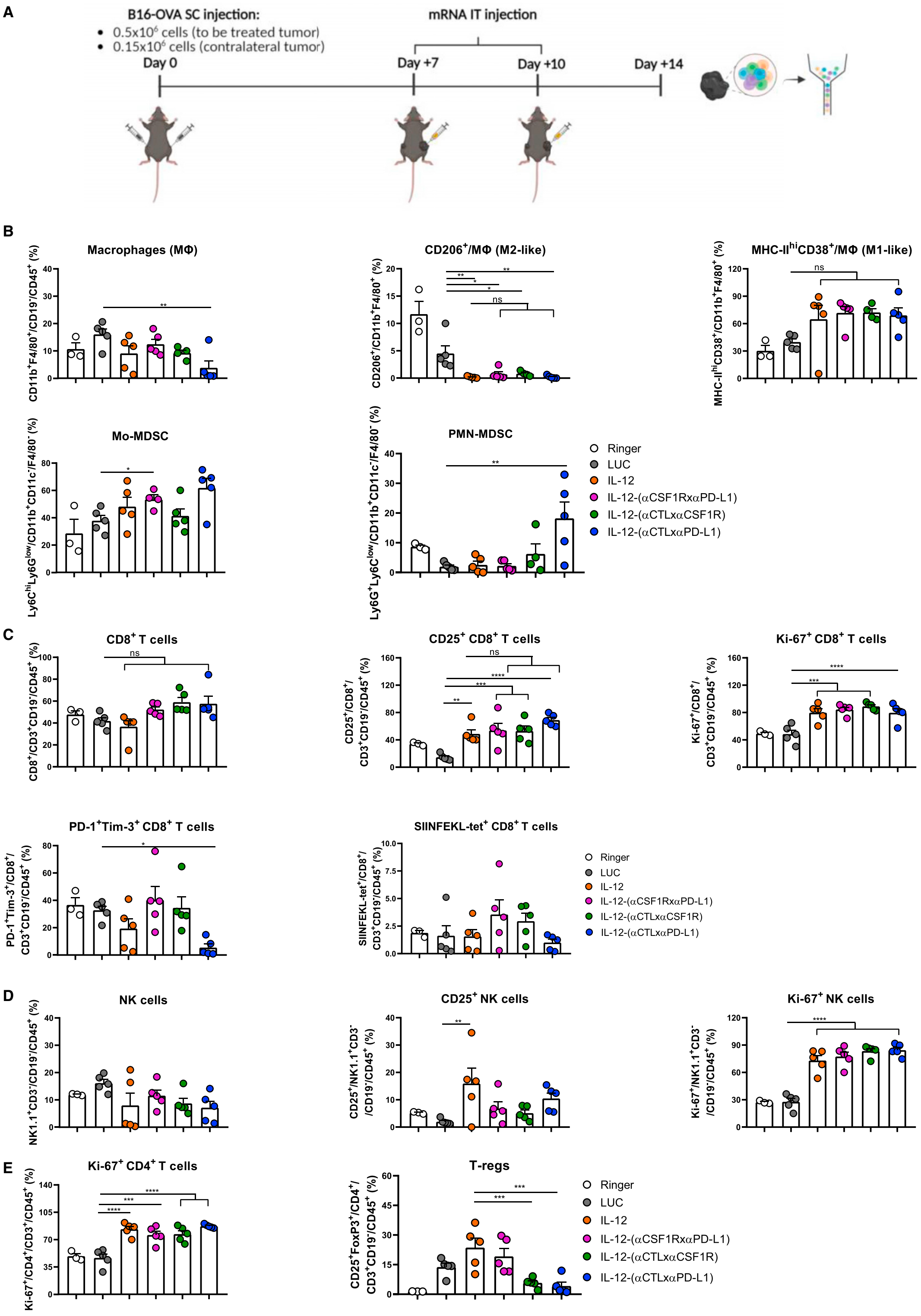

Figure 4. IL-12 fused to the diabodies maintains its bioactivity *in vivo*

(A) The scheme summarizes the treatment regimen and the timeline of the flow cytometry analysis, whose results are presented in (B)–(E). B16-OVA-bearing mice (n = 3–5) received 2 doses of the indicated mRNAs on day 7 and day 10 after tumor injection and were sacrificed 4 days after the last treatment (0.5 μg IL-12-encoding mRNA and

(legend continued on next page)

$CD8^+$ T lymphocytes positively stained for IFN-γ and GrB, compared with the LUC condition. Thus, a modest but measurable increase in the cytotoxic activity in the contralateral untreated tumor was detected. To clarify whether the intratumoral treatment induces a systemic antitumor immunity, the splenocytes of the same mice were *ex vivo* re-stimulated with the cognate antigens and assayed in IFN-γ ELISpot assays. As shown in Figure S8A, both tethered and untethered IL-12 were able to elicit $CD8^+$ T cell-mediated immune responses of comparable intensity. To confirm these findings, similar ELISpot assays were performed in the MC38 colon carcinoma tumor model, and an *in vivo* killing assay was conducted in the B16-OVA model. In both experiments, tethered and untethered IL-12 mRNA-based intratumorally delivered treatments were able to elicit antigen-specific $CD8^+$ T cell-mediated systemic immunity toward antigens of the tumor cell lines, compared with irrelevant mRNA-injected mice (Figures S8A–S8C).

Next, we sought to investigate the potential activity of the chimeric mRNA-encoded constructs to attain antitumor efficacy. To this end, we locally administered to B16-OVA bilaterally engrafted mice a three-dose regimen of LUC-, IL-12-, or IL-12 diabody-encoding mRNAs (Figure 5A). In the injected lesion, all IL-12-fused diabodies achieved a similarly potent antitumor effect as non-chimerized IL-12. In at least 50% of cases, the treated lesion became undetectable (Figure 5B). Interestingly, we also observed a significant delay in the tumor growth rate in the contralateral untreated lesions when these were treated with IL-12, IL-12-(αCSF1RxαPD-L1), and IL-12-(αCTLxαPD-L1) (Figure 5C). The delayed tumor growth translated into a significantly prolonged overall survival mediated by the injection of all the IL-12-bearing constructs (Figure 5D). Of note, the IL-12 moiety was an essential requirement for the antitumor effects, as the tumor growth and mice survival after injection of the IL-12-devoid bispecific diabody were comparable with those in the LUC-mRNA-injected mice, whereas fusion with IL-12 significantly increased overall survival compared with the non-chimerized construct (Figures S9A–S9C). Importantly, the intratumoral administration of mRNAs encoding the different IL-12 constructs was safe. Mice showed no signs of distress, and they did not lose weight at any of the evaluated time points (Figure S10A).

The mRNA-based therapy was then evaluated in a model of bilateral MC38 colorectal cancer (Figure 6A). These experiments confirmed the potent antitumor effect exerted by the IL-12-carrying constructs on the treated lesion and a less prominent effect, but still measurable activity, in the contralateral uninjected lesion (Figures 6B and 6C). Interestingly, only the IL-12-(αCSF1RxαPD-L1) and the IL-12-(αCTLxαPD-L1) constructs led to a statistically significant effect (Figure 6D). As observed in the B16-OVA model, the administration of the treatment was safe, and no weight loss was found in any case (Figure S10B).

mRNA-encoded IL-12 diabodies tether the heterodimeric cytokine to myeloid populations infiltrating the tumors thereby limiting systemic leakage

In order to investigate if IL-12 diabodies were able to anchor IL-12 to the tumor niche after *in vivo* delivery of the mRNAs, we designed an experiment assessing the tethering of these molecules *in vivo* to myeloid cells nesting in the engrafted tumors. To this end, B16-OVA-subcutaneously implanted mice were intratumorally injected with the different mRNAs. 19 h post-treatment delivery mice were sacrificed, and cell suspensions from the excised tumors were stained with fluorochrome-labeled antibodies in order to characterize if the tumor-infiltrating myeloid cells were decorated with IL-12 on their surface (Figure 7A). In line with the results observed in the *ex vivo* tethering experiment (Figure 3B), the three diabodies were able to tether IL-12 to tumor-infiltrating macrophages. Strikingly, only IL-12-(αCSF1RxαPD-L1) and IL-12-(αCTLxαPD-L1) diabodies led to a significantly higher linkage of the cytokine to intratumoral myeloid leukocytes compared with tumors from mice injected with IL-12 alone. These two diabodies were also able to anchor IL-12 to mo-MDSC and PMN-MDSC populations infiltrating the tumors (Figure 7B).

To evaluate the impact of the tethering ability on systemic exposure, we evaluated using ELISA the IL-12p70 levels in the tumor homogenates and sera of mice 6 h after intratumoral mRNA injection. When IL-12 was bound to the targeting diabodies, systemic exposure to cytokine was significantly decreased in contrast to the similar levels attained in the tumor microenvironment 6 h after mRNA delivery (Figures 7C and 7D). We could not detect any IL-12p70 in serum from any mice treated with the mono- or bispecific IL-12 diabody mRNAs up to 96 h after mRNA delivery (Figure 7E). In line with these results, circulating levels of IFN-γ were higher after IL-12 mRNA administration compared with the targeted chimeric constructs. IFN-γ concentrations peaked 24 h post-inoculation and remained elevated up to 72 h after treatment (Figure 7F). In the case of the mice injected with the IL-12 diabodies, we were able to detect low levels IFN-γ only in some of the mice.

These results demonstrate that the diabody chimeras prevented the systemic leakage of IL-12 and, as a consequence, avoided the increase of IFN-γ levels in circulation while conserving therapeutic efficacy.

10 μg of the mRNAs encoding the diabodies were used per each dose). Immune cells of the excised tumors were phenotypically characterized by multi-color flow cytometry analysis. (B) Macrophage, mo-MDSC, and PMN-MDSC populations were gated as in Figure 3B. The intracellular expression of CD206 and the surface co-expression of CD38 and MHC-II were assessed among the macrophages. (C) The expression of CD25, Ki-67, PD-1, Tim-3, and the percentage of tetramer-positive cells among $CD8^+$ T cells was assessed. $CD8^+$ T cells were gated as viable $CD45^+CD19^-CD3^+CD4^-CD8^+$. (D) The expression of Ki-67 and CD25 among NK cells was measured. NK cells were gated as viable $CD45^+CD19^-CD3^-NK1.1^+$. (E) Ki-67 in $CD4^+$ T cells, which were defined as $CD45^+CD19^-CD3^+CD8^-CD4^+$. Treg population was defined as $CD45^+CD19^-CD3^+CD8^-CD4^+CD25^+Foxp3^+$. The gating strategy used is shown in Figures S4A and S4B. Data are shown as mean ± SEM. Statistical significance was determined using one-way ANOVA followed by Šidák's multiple-comparisons tests (*p < 0.05, **p < 0.001, ***p < 0.001, and ****p < 0.0001).

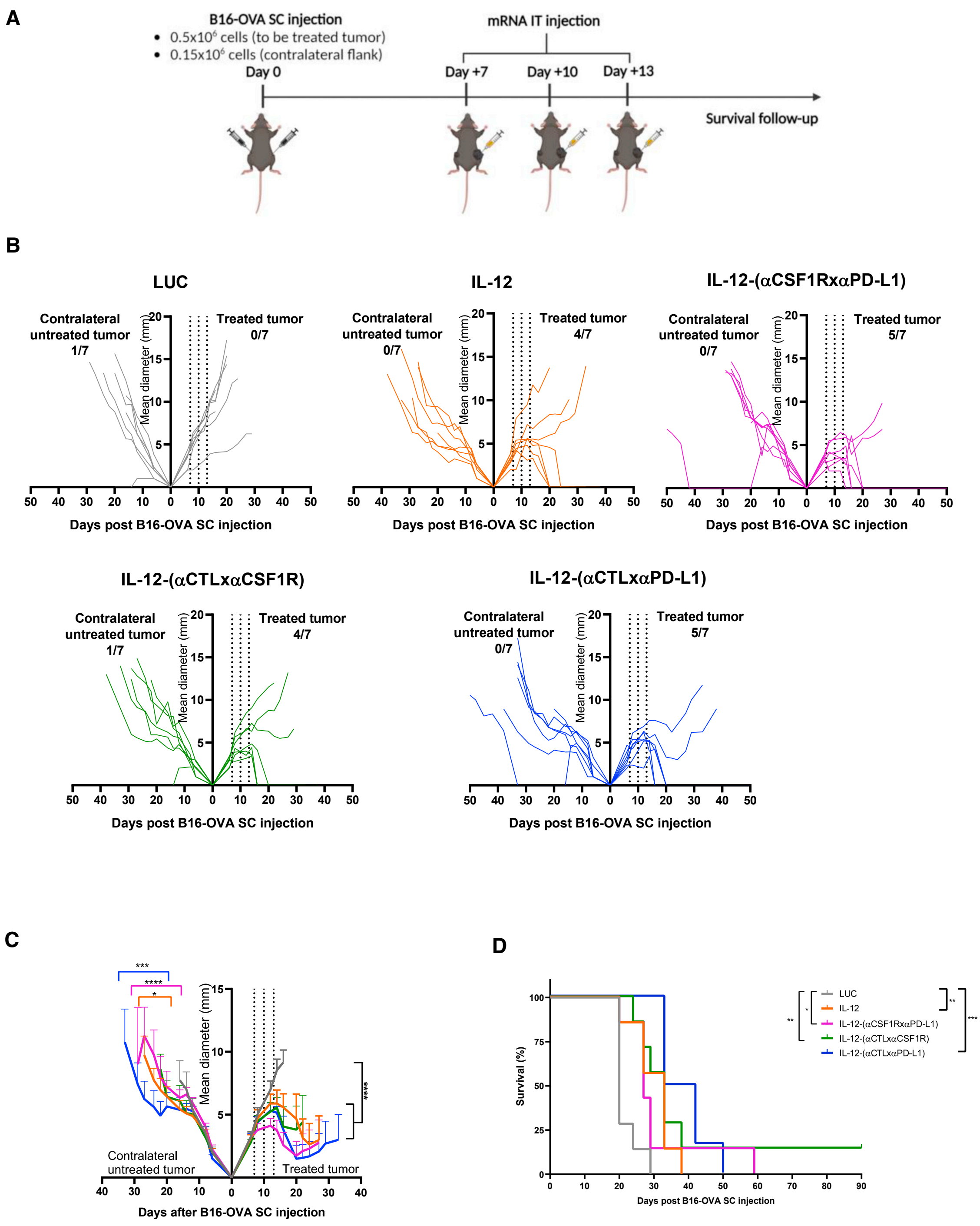

Figure 5. IL-12-fused diabodies exert a strong antitumor efficacy in a bilateral B16-OVA tumor model

(A) Schematic representation of the experimental procedure. Mice received 3 doses of 0.5 μg IL-12-encoding mRNA or 10 μg of the mRNAs encoding LUC or the IL-12-fused diabodies. (B) Individual follow-up of tumor sizes with the fraction of cured tumors is presented. (C) Cumulative data of (B) with statistical comparison. (D) Mice survival follow-up is shown (n = 7/group). Longitudinal data were fitted to a third-order polynomial equation and compared using an extra sum-of-squares F test for (C). Survival data in (D) were analyzed using log rank (Mantel-Cox) tests (**p < 0.01, ***p < 0.001, and ****p < 0.0001).

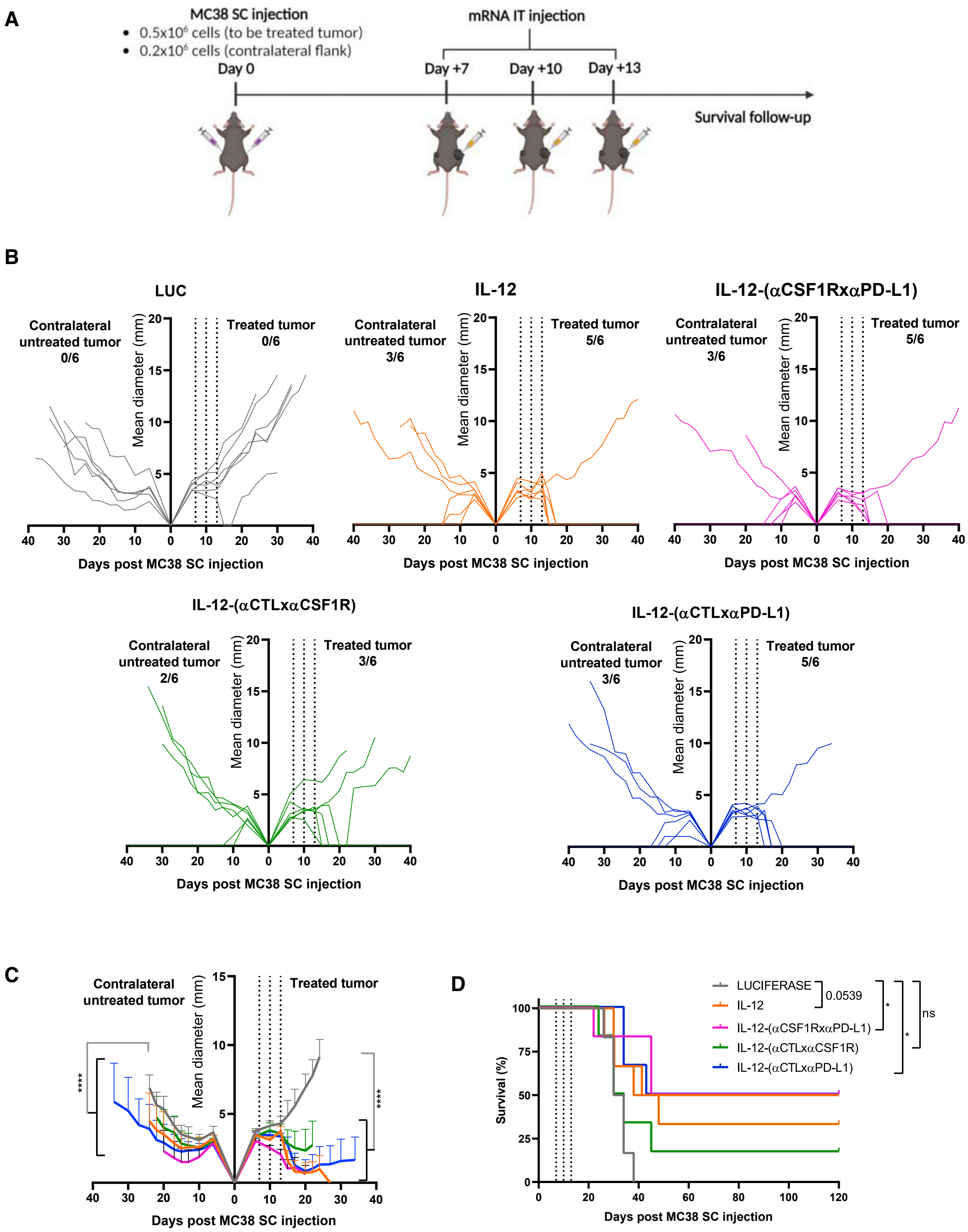

Figure 6. Antitumor effects of IL-12-fused diabodies are replicated in a bilateral MC38 tumor model

(A) Schematic representation of the experimental procedure. Mice received 3 doses of 0.5 μg IL-12-encoding mRNA or 10 μg of the mRNAs encoding LUC or the diabodies. (B) Individual follow-up of tumor sizes with the fraction of cured tumors is presented. (C) Cumulative data of (B) with statistical comparison. (D) Mice survival follow-up is shown (n = 6/group). Longitudinal data were fitted to a third-order polynomial equation and compared using an extra sum-of-squares F test for (C). Survival data in (D) were analyzed using log rank (Mantel-Cox) tests (*p < 0.05 and ****p < 0.0001).

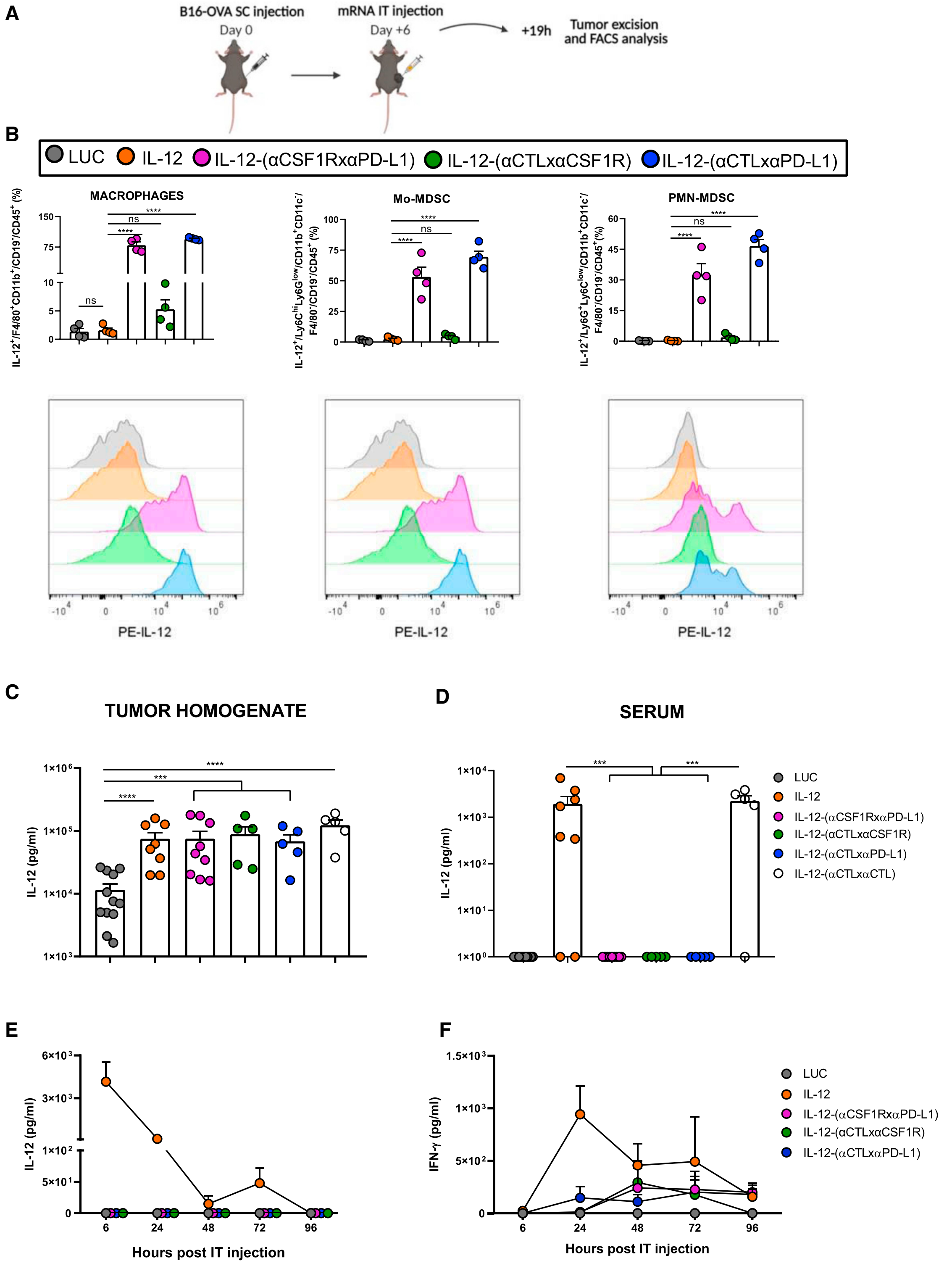

Figure 7. Diabodies tether the fused IL-12 to the tumor-infiltrating myeloid populations avoiding systemic leakage

(A) Scheme summarizing the flow cytometry experiment of (B). Subcutaneously implanted B16-OVA-bearing mice (n = 4/group) were IT delivered with naked mRNAs. 19 h post-injection, tumors were excised, processed, and surface-stained with the same antibody cocktail as in Figure 3. (B) Each graph represents the percentage of the indicated populations with IL-12 anchored on their surface, demonstrating the *in vivo* tethering ability of IL-12-fused diabodies. The histogram overlay shows the flow

(legend continued on next page)

Analyses of previously published single-cell RNA sequencing (scRNA-seq) of tumor tissue samples from melanoma and colorectal cancer patients showed how both CSF1R and PD-L1 are particularly enriched in the myeloid populations infiltrating the human tumors (Figure S11). Altogether, this evidence suggests that our strategy of tethering IL-12 to the tumor niche is effective while broadening the safety margins of local immunotherapy based on IL-12.

DISCUSSION

The main purpose of the present study has been to evaluate new strategies to overcome some of the limitations of IL-12-based gene therapy applications. Indeed, the early clinical trials using the systemic infusion of recombinant IL-12 demonstrated the poor tolerability and limited efficacy of the treatment mostly due to IFN-γ-related toxicity.[14,15] This has prompted the development of a plethora of methodologies to make the most of antitumor activities mediated by IL-12, as reviewed by Cirella *et al.*[16,33] Our first goal consisted of evaluating the feasibility of intratumoral delivery of naked mRNA encoding IL-12 in Ringer's lactate solution and the subsequent antitumor response. We intratumorally delivered IL-12-encoding mRNA into subcutaneously implanted tumors and observed how doses ranging from 10 to 0.5 μg of the mRNA were correctly translated and secreted in the tumor niche. Taking advantage of the intrinsic short half-life of the mRNA molecule that allows us to inject the therapeutic molecule repeatedly, we used a three-dose regimen injected every 72 h to treat the tumors. As little as 0.5 μg were able to attain potent cancer control in the treated lesion and some delay in growth of the distant uninjected tumor. These effects were accompanied by detectable levels of IL-12p70 and IFN-γ in peripheral blood. To the end of limiting IL-12 leakage to the circulation, we designed fusion molecules to retain IL-12 inside the tumor microenvironment while preserving its antitumor efficacy. For this purpose, we produced IL-12-fused diabodies, trimeric molecules encompassing the heterodimeric cytokine, and two targeting antibody-based arms. The first chosen target was CSF1R. Upon ligand binding, this receptor is critical for the differentiation and survival of the mononuclear phagocytes, being of particular relevance in macrophages.[34] In the cancer setting, high levels of its ligand CSF-1 lead to tumor-associated macrophage recruitment and to polarization toward a tumor-supporting phenotype.[35–37] Many clinical trials are evaluating small molecules or mAbs blocking CSF-1/CSF1R signaling.[38] The choice of the second target was driven by the knowledge that tumor and stromal cells can deploy adaptive immune resistance mechanisms to escape the antitumor effects mediated by the IL-12-induced IFN-γ. In fact, IFN-γ leads to increased expression of the PD-L1 protein on tumor cells and myeloid cells, which in turn can bind to PD-1 on T cells to suppress antitumor immunity.[39] Indeed, many preclinical and clinical studies exploit the synergy between IL-12-dependent antitumor efficacy and the blockade of the PD-1/PD-L1 axis (NCT03871348).[40,41] Aside of retention in the tumor microenvironment, the diabodies may target IL-12 to myeloid cells that may either directly sense IL-12 or functionally interact with T and NK lymphocytes.

The generated tumor-tethering IL-12 diabodies, either those binding to both targets or each single target, avoided systemic leakage of IL-12 compared with IL-12 alone or IL-12-fused to an irrelevant diabody. All the constructs showed bioactivity *in vitro* and were correctly translated *in vivo*, even though the translation efficiency of the immunocytokines proved to be much lower compared with IL-12, perhaps as a result of being longer mRNAs. Furthermore, IL-12 diabody constructs maintained IL-12 antitumor bioactivity and were functional at reprogramming the leukocyte composition of the tumor microenvironment. In addition to tethering the cytokine in the tumor milieu, IL-12-bispecific immunocytokine retained the capacity of the untethered cytokine to generate a local and systemic $CD8^+$ T cell-dependent immune response specific for the antigens of the tumor cell lines achieving modest, though measurable, abscopal effect on uninjected tumor lesions.

Many optimizations could be further incorporated, such as improvements in mRNA translation and stability, codon optimization, use of different linker sequences, exploring other Fc-devoid antibody formats, or introducing sequence modifications to avoid potential undesired secondary structures.[42] The delivery method could also be refined. Most of the mRNA therapeutics currently under development rely on lipid nanoparticles for delivery.[43] In the present study, we used naked mRNAs in Ringer's lactate saline solution as already performed preclinically[19,20,27–29] and in the clinic with a cocktail of cytokine-encoding mRNAs, including scIL-12 (NCT03871348). This is considered a cheaper alternative strategy that has the advantage of minimizing the potential for off-target effects and adverse reactions associated with the unnatural lipid formulation. We demonstrated that sufficient mRNA translation occurs to provide a therapeutic benefit. Nonetheless, exploring other lipid-based delivery strategies to further increase mRNA uptake represents an exciting option.[44,45]

Altogether, our work outlines a promising platform of chimeric constructs that can be exploited to generate therapeutic mRNAs. Local intratumoral delivery in a lipid-free saline solution results in the translation of the trimeric constructs, maintaining the functionality

cytometry analysis of IL-12 surface detection of one representative sample of each condition. (C) Mice were injected with 0.5 × 10^6 B16-OVA cells subcutaneously in the right flank. Ten days post-tumor inoculation, the different indicated mRNAs were intratumorally injected. Six hours later, IL-12p70 cytokine was quantified using ELISA in the homogenates of the excised tumors and in sera (LUC, n = 12; IL-12, n = 8; IL-12-[αCSF1RxαPD-L1], n = 9; IL-12-[αCTLxαPD-L1], IL-12-[αCTLxαPD-L1], and IL-12-[αCTLxαCTL], n = 5). (D) In a different subset of mice, sera were collected 6, 24, 48, 72, and 96 h after mRNA administration (0.5 μg IL-12-encoding mRNA or 10 μg of each of the IL-12 diabody-encoding mRNAs), and IL-12 and IFN-γ levels were determined using ELISA (n = 4 or 5). Data are shown as mean ± SEM. Statistical significance was determined using one-way ANOVA followed by Šidák's multiple-comparison tests in (B) and using Dunnett's multiple-comparison tests of log-transformed data in (C) (***$p < 0.001$ and ****$p < 0.0001$).

of each moiety. These modules of mRNA-encoded immunocytokines hold promise for local treatment of cancer lesions and are also a step forward in the search for systemic immune-mediated effects.

MATERIALS AND METHODS

Animal handling

Experiments involving mice were approved by the Ethics Committee of the University of Navarra (R-080-19GN). We have complied with all relevant ethical regulations for animal testing and research. Experiments were performed in 6- to 10-week-old C57BL/6J mice that were purchased from Harlan Laboratories (Barcelona, Spain).

Cell lines and tumor mouse models

The B16-OVA melanoma cell line and MC38 colon carcinoma cells were a kind gift from Dr. Lieping Chen (Yale University, New Haven, CT) in November 2001. Both cell lines were maintained in complete RPMI medium: RPMI 1640 medium with GlutaMAX (Gibco, Waltham, MA) supplemented with 10% fetal bovine serum (FBS) (Sigma-Aldrich, St. Louis, MO), 100 IU/mL penicillin and 100 μg/mL streptomycin (Gibco) and 50 μM 2-mercaptoethanol (Gibco). In the case of B16-OVA cells, medium was supplemented with 0.4 mg/mL of G418 (Sigma-Aldrich). All cell lines were routinely tested for mycoplasma contamination using the MycoAlert Mycoplasma Detection Kit (Lonza). For the injection in the right flank (the tumor to be treated), both cell lines were resuspended at a final concentration of 0.5×10^6 cells and delivered in 100 μL of PBS per mouse. B16-OVA cells were resuspended at a final concentration of 0.15×10^6 in 100 μL of PBS per mouse for the injection in the contralateral flank. MC38 cells were resuspended at a final concentration of 0.2×10^6 in 100 μL of PBS.

Plasmids and mRNA synthesis by *in vitro* transcription

The scIL-12 sequence consists of the murine IL-12p40 subunit, including its own signal peptide, but depleted of the stop codon, followed by a flexible linker of 15 amino acids $(GGGGS)_3$ followed by the murine IL-12p35 subunit deprived of its signal peptide and followed by a stop codon.[26,46,47] All the IL-12 diabodies had the following structure: (scIL-12)-(GSADGG)-(Variable Heavy chain a [VHa])-(GGSGG)-(Variable Light chain b [VLb])-(SSSSGSSSSGSS SSG)-(VHb)-(GSADGG)-(VLa). The 15 amino acid linker between the two sets of VH and VL sequences is long enough to allow pairing of the VH and VL domains of the first set (a) with the complementary domains of the second set (b). In contrast, the VH-VL sequences within each set are connected by a non-flexible linker of 5 amino acids, which is not sufficiently long to allow pairing of the VH and VL domains. The variable region sequences targeting CSF1R were obtained by sequencing the complementarity-determining regions (CDRs) of the AFS98 hybridoma which produces a rat IgG2a anti-mouse CSF1R antibody. The avelumab antibody is a fully human anti-PD-L1 IgG1-λ antibody that binds to human and murine PD-L1 and blocks its interaction with PD-1, and its variable region sequences were obtained from the DrugBank online database. For the irrelevant IL-12 diabody, the VH and VL sequences of the B12 antibody were obtained by sequencing the CDRs of a hybridoma coding for a murine IgG1-κ neutralizing antibody against a glycoprotein of human HIV-1, a non-relevant antigen in murine *in vivo* models. The Firefly LUC and GFP protein sequences were obtained from the UniProt database (#P08659 and #C5MKY7, respectively). All the sequences were codon-optimized for *Mus musculus* and synthesized and cloned by GenScript (Nanjing, China) in a pUC57 backbone. The sequence upstream of the first codon of the open reading frame (ORF) comprised the T7 promoter and a Kozak sequence. The stop codon was followed by 2 sequential β-globin 3′UTR cloned head to tail and a 90–120 poly(A) tail.[48] The sequence of the generated constructs was verified by direct sequencing and by double restriction enzyme digestion. Linearized DNA (1–2 μg) was subjected to *in vitro* transcription (IVT), and to post-transcriptional capping and poly-adenylation (which was carried out to ensure an optimal size of the poly(A) tail) using the T7 mScript_Standard mRNA Production System (Cellscript, Madison, WI), as previously described.[47] The purified mRNA was eluted in RNase-free water at 1–2 mg/mL and stored at −80°C.

Luminescence detection with PhotonIMAGER

Twelve days after B16-OVA subcutaneous tumor inoculation, 10 μg of LUC mRNA were brought to room temperature and injected in a final volume of 50 μL of Ringer's lactate solution intratumorally in each mouse. At the time points indicated in the results section, *in vivo* bioluminescence was detected in order to study the kinetics of mRNA expression *in vivo*. To this end, 100 μL of luciferin (Promega, Madison, Wisconsin) (20 mg/mL) were administered intraperitoneally. After 5 min, bioluminescence was detected using PhotonIMAGER (Biospace Lab, Paris, France). The data were analyzed using M3 Vision software.

Design of *in vivo* experiments with intratumoral delivered naked mRNAs

In the experiments performed in the bilateral B16-OVA tumor model, the volume of the treated tumors on the day of the first dose ranged between 50 and 155 mm^3, and the size of contralateral tumors ranged between 19 and 72 mm^3. In the case of MC38 tumor model, the treated tumor average volumes were 58 mm^3, and the untreated were 31 mm^3. For the survival follow-up experiments, 5 μL of each mRNA were formulated in 45 μL of Ringer's lactate solution and injected into the lesion implanted in the right flank 7, 10, and 13 days after tumor inoculation. For the study of the kinetics of expression of the mRNAs encoding IL-12 and IL-12 diabodies, mice were intratumorally injected seven days post-B16-OVA tumor inoculation. 0.5 μg IL-12-encoding mRNA was found to give comparable IL-12p70 levels in the tumor niche as 10 μg IL-12-(αCSF1RxαPD-L1) and these doses were used for the *in vivo* experiments, unless otherwise specified. For the analysis of the *in vivo* tethering capacity of the chimeric constructs, 1.5 μg IL-12-encoding mRNA and 30 μg IL-12 diabody-encoding mRNAs were intratumorally injected six days after B16-OVA subcutaneous tumor inoculation in a final volume of 100 μL. Nineteen hours post-delivery, tumors were excised and processed as described in the next section. For the characterization of the tumor immune microenvironment, B16-OVA-bearing mice were injected

with the mRNAs on days 7 and 10 after tumor injection. Tumors were excised and processed four days after the second mRNA dose.

Sample processing

For mice organ processing, animals were euthanized with CO_2. To determine the protein levels of IL-12 and IFN-γ in the tumor homogenates, subcutaneously implanted tumors were excised and quickly homogenized by mechanical disruption with a pestle in a pre-chilled tube containing 200 μL of PBS with cOmplete protease inhibitor cocktail (#11836145001; Roche). After 15 min centrifugation at maximum speed, the supernatants were isolated and stored at −80°C until further analysis with ELISA.

For flow cytometry characterization of the immune cells after treatment, and for studying the *ex vivo* and *in vivo* tethering of the chimeric molecules, the tumors were excised, cut into small pieces (2–5 mm), and incubated with 400 MandL/ml of collagenase and 50 μg/mL of DNase (Roche) for 30 min at 37°C. Spleens were isolated from the mice, and all the specimens were disrupted by mechanical force through a 70 μm filter and abundantly washed with PBS or with staining buffer in the case of tumor samples, as it contains EDTA, which blocks the collagenase activity. Splenocytes and tumor samples were incubated with ammonium-chloride-potassium (ACK) lysing buffer (Gibco) for 2 min to lyse erythrocytes. The ACK buffer was diluted with an excess volume of complete RPMI medium, cells were washed, resuspended in PBS, and counted. Single-splenocyte suspensions were kept at 4°C until further analysis. Tumor-infiltrating leukocytes were isolated by centrifugation of the tumor cell pellet in 35% Percoll (GE Healthcare), washed, resuspended in PBS, and kept at 4°C until further analysis.

Ex vivo transfection and western blot

B16-OVA were seeded in multi-well 12 or multi-well 6 plates one day before transfection. Once they reached 70%–80% confluency, they were transfected with 1–2.5 μg of each mRNA formulated with the TransIT-mRNA Transfection kit (Mirus Bio), following the manufacturer's instructions. For cytokine detection in the supernatants, cell cultures were harvested at the indicated time points, centrifugated, and the supernatants stored at −80°C until further analysis.

For Western Blot technique, 24 h after transfection supernatants were discarded, wells were gently washed with PBS, and cells were lysed by adding 200 μL of RIPA buffer enriched with protease inhibitors (Na_3VO_4 [#S6508; Sigma-Aldrich], NaF [#S7920; Sigma-Aldrich], cOmplete [Roche]) to each well. Adherent cells were scraped and moved to a pre-chilled tube and left on ice for 30 min. Following 30 min spinning at 4°C at maximum speed, protein lysates were recovered and stored at −80°C until further analysis. Protein lysates were spectrophotometrically quantified using the Bio-Rad Protein Assay Dye (#5000006), and 20 μg of each sample were prepared in loading dye + 2.5% β-mercaptoethanol (#M6250; Sigma-Aldrich) and was run in a 10% polyacrylamide gel sodium dodecyl sulfate-polyacrylamide gel electrophoresis (SDS-PAGE). The molecular weight of each translated construct was checked by immunoblotting the membrane with the following primary antibodies O/N at 4°C: rat anti-mouse IL-12 p40 antibody (#BE0051; BioXcell), rabbit anti-mouse actin antibody (#A2066; Sigma-Aldrich). Subsequently, membranes were incubated with HRP-linked secondary antibodies for 1 h at room temperature: anti-rabbit (#8085S; Cell Signaling Technology, Danvers, MA), anti-rat (#NA935; Amersham).

ELISA determination of cytokine concentrations and *in vitro* binding assays

IFN-γ and IL-12 protein levels in the sera and tumor homogenates of mice were assayed by ELISA (#555138 and #555256; BD Biosciences, San Diego, CA), following the manufacturer's instructions. To check the bioactivity of IL-12 deriving from translation of mRNAs encoding IL-12-carrying constructs, splenocytes were isolated from mice, pre-activated with plate-bound anti-CD3 (1 μg/mL) (#100314; BioLegend, San Diego, CA) for 24 h and incubated for two days with supernatants collected 72 h after B16-OVA *in vitro* transfection. The triggered production of IFN-γ was measured by an ELISA. Data were analyzed and interpolated in the standard curve values using GraphPad Prism. To check the binding capacity of each targeting arm to its specific target, an *in vitro* binding assay was designed. Each well of a Nunc MaxiSorp 96-well plate was coated with 2 μg/mL murine CSF1R (#CSR-M52E7; Acrobiosystems) or 1 μg/mL of murine PD-L1 (#50010-M08H; Sinobiological) recombinant proteins in 50 μL of PBS ON. The following day, after three washes with PBS 0.05% Tween 20 (#P7949; Sigma-Aldrich), unspecific binding was blocked by adding 200 μL of assay diluent (PBS 10% FBS) to each well for 2 h at room temperature. After three washes, the supernatants recovered from *in vitro* transfected cells were added to the wells and incubated for 2 h at room temperature. Plates were washed five times and incubated with the HRP-linked detection antibody of the IL-12 ELISA (#555256), following the manufacturer's instructions, for 1 h at room temperature. Each plate was washed 7 times, the TMB substrate solution (#555214; BD OptEIA) was added, and after 5–15 min, depending on the experiment, sulfuric acid was added to each well, and optical density was measured at 450 nm.

Flow cytometry

For flow cytometry staining, cells obtained from tumor samples were stained using PromoFluor 840 (#840301; PromoCell, Heidelberg, Germany) for 5 min at room temperature. After being washed with staining buffer (PBS + 2% FBS, 2 mM EDTA, 1% 100 IU/mL penicillin, and 100 μg/mL streptomycin (Gibco)), cells were treated with FcR-Block (anti-CD16/32 clone 93; BioLegend #101302) for 10 min at 4°C. For the *in vivo* tethering experiments and tumor immune microenvironment characterization, cells were subsequently surface-stained with fluorochrome-labeled antibodies for 20 min at 4°C protected from light. In the case of the *ex vivo* tethering experiment, an intermediate step was performed: the single-cell suspensions of excised tumors were incubated with 100 μL of medium collected 72 h after B16-OVA *in vitro* transfection for 20 min at 4°C. The supernatants of all conditions were used at the same IL-12 dose (460 ng) as ascertained by an ELISA. Cells were washed twice with

staining buffer and subsequently surface-stained with fluorochrome-labeled antibodies for 20 min at 4°C protected from light. All the antibodies used in the current work were purchased from BioLegend (unless otherwise specified): PerCPeFluor710-CD3 (#46-0032-80; eBioscience, San Diego, CA); BUV395-CD8 (#563786; BD Biosciences); BUV496-CD4 (#564667; BD Biosciences); BV650-CD19 (#115541); BV605-PD-1 (#135220); PE-Dazzle594-NK1.1 (#108747); APC-CD25 (#102012); PE-Cy7-CD45.2 (#109829); APC/Fire750-Tim-3 (#134018); FITC-Ly6C (#128006); PerCP5.5-CD45.2 (#109828); APC-eFluor780-MHC-II (I-A/I-E) (#47-5321-82; eBioscience); PE-Dazzle594-CD38 (#102729); BV421-F4/80 (#123131); BV510-LY6G (#127633); BV785-TCRb (#109249); BUV395-CD11b (#563553; BD Biosciences); APC-F4/80 (#123116), BV605-CD11c (#117333); and IL-12/IL-23 p40 (#505203).

When required, cells were intracellularly stained. The Foxp3/TF staining buffer kit (eBioscience) was used according to manufacturer's instructions for the intracellular staining with BV421-FoxP3 (#126419) and AF488-Ki-67 (#558616; BD Biosciences). The Fixation/Permeablization Kit (BD Biosciences) was used for the intracellular staining with PE-Cy7-CD206 (#141719), AF647-IFN-γ (#505816), and FITC-GrB (#11-8898-82; eBioscience). In the case of the experiment were the intracellular staining of IFN-γ and GrB was performed, all the *ex vivo* steps (from DNase/collagenase incubation up to fixation/permeabilization step) were performed by adding to each buffer the fixation Brefeldin A Solution 1 X (#420601).

To assess the IL-12-dependent phosphorylation of STAT-4, murine splenocytes were pre-activated O/N using plate-bound anti-CD3 (1 μg/mL) (#100314; BioLegend) and soluble anti-CD28 (#122021; BioLegend; 1 μg/mL). Twenty-four hours later, 3 million pre-activated splenocytes were incubated for 1 h at 37°C with the conditioned media from mRNA-transfected B16-OVA cells, containing 400 ng IL-12 in all conditions, as previously assessed by a p70/IL-12 ELISA sandwich assay. Subsequently, cells were washed and the cell pellet was resuspended in BD Cytofix Fixation Buffer (#554655 BD; Biosciences) for 15 min at 37°C. After extensive washing, cells were permeabilized using pre-chilled BD Phosflow Perm Buffer III for 30 min at 4°C and then stained with Phospho-STAT-4 (Tyr693) (#17-9044-42; Invitrogen) and PerCP-CD45 (#103130) for 45 min at RT. In all cases, after extensive washing, cells were resuspended in 150–200 μL staining buffer and immediately assayed in a CytoFLEX flow cytometer (Beckman Coulter, Brea, CA).

ELISpot and *in vivo* killing

For ELISpot experiments, mice splenocytes were isolated 48 h after the second mRNA dose and erythrocytes lysed as described above. In the case of B16-OVA-bearing mice, 0.8×10^6 splenocytes were stimulated with 0.001 mg/mL $OVA_{257–264}$ peptide (Invivogen, San Diego, CA) or with medium alone, whereas in the experiment of MC38-bearing mice, 1×10^6 splenocytes were stimulated with 0.01 mg/mL KSPWFTTL peptide or with medium alone. In both cases, splenocytes were incubated on an IFN-γ ELISpot plate (BD Biosciences) for 24 h. After extensive washing, plates were incubated with the biotinylated detection antibody for 2 h at RT. The wells were washed and incubated with the Streptavidin-HRP solution for 1 h at RT. 1X AEC Substrate Solution was added to the wells for 10 min, and then these were soaked in DI water to stop the reaction. Plates were left drying O/N and spot-forming cells were automatically counted using an ImmunoSpot counter (CTL-ImmunoSpot, Bonn, Germany). In order to measure *in vivo* killing capability of $CD8^+$ T cells, single-cell suspensions of splenocytes were obtained from naive mice. Half of the splenocytes were pulsed for 30 min at 37°C with 0.01 mg/mL $OVA_{257–264}$ peptide and the other half was incubated with the same volume of PBS only. Tubes were gently mixed every 10 min. After extensive washing, the two populations were stained with 5 mM and 0.5 mM of CFSE dye (#565082; BD Biosciences), respectively, and were mixed in a 1:1 ratio in order to retro-orbitally inject 10×10^6 cells per mouse 24 h after the third mRNA dose. Twenty hours post-transfer of the stained cells, spleens were isolated, processed as described above and single-cell suspensions were analyzed using flow cytometry. To calculate the percentage of specific lysis of the target cell population, the following equation was used for each treated mouse, compared with naive control: %specific lysis = $100 - [100 \times (\%CFSE^{high}$ immunized mouse/% $CFSE^{low}$ immunized mouse)/(% $CFSE^{high}$ naive mouse/% $CFSE^{low}$ naive mouse).

Analysis of scRNA-seq data

For scRNA-seq expression analysis, we selected a cohort of 31 patients with melanoma tumors[49] obtained from GEO datasets under accession number GSE115978 and experiment GSE132465 with colorectal cancer from 23 different tumors.[50] Subsequent analysis was performed in R/Bioconductor. First, we selected only the tumor fraction of the datasets. Subsequently, raw counts were normalized on the basis of their annotated cell type provided by the contributors following the OSCA pipeline.[51] Uniform manifold approximation and projection (UMAP) figures and expression dot plots were generated with the functions provided by the Seurat package.[52]

Statistical analysis

Prism version 8.2.1 software (GraphPad Software, San Diego, CA) was used for statistical analysis. Data were analyzed using two-tailed unpaired t tests (Mann-Whitney), and by one-way analysis of variance followed by Šidák's or Dunnett's multiple-comparisons tests. Survival analysis was performed using the Kaplan–Meier method. The log rank test was used to compare the curves statistically. p values <0.05 were considered to indicate statistical significance.

DATA AND CODE AVAILABILITY

The authors confirm that the data supporting the findings of this study are available within the article and its supplementary materials.

SUPPLEMENTAL INFORMATION

Supplemental information can be found online at https://doi.org/10.1016/j.omtn.2023.07.020.

ACKNOWLEDGMENTS

The authors thank Dr. Paul Miller for English editing and Cristina Lopez and Cibeles Pinto for the excellent secretarial assistance. We would like to acknowledge both the flow cytometry facility (Diego Alignani) and the imaging facility of CIMA center. We thank Dr. Natasa Vukovic for the helpful scientific discussions. C.A.D.T. is grateful to Ruben Jurado Gomez for teaching the power of mindful breathing to focus and to help improve work-life balance. Figures contain elements from BioRender (https://biorender.com). This work was supported by Instituto de Salud Carlos III (PI22/00147, PI19/01128, and PI20/00002) co-financed by Fondos Feder. This project has received funding from the European Union's Horizon 2020 research and innovation program under the Marie Skłodowska-Curie grant agreement 765394 and Gobierno de Navarra Proyecto ARNMUNE (0011–1411-2023). Work produced with the support of a 2022 Leonardo Grant for Researchers and Cultural Creators (BBVA Foundation). M.A. was supported by a Spanish Association Against Cancer's Investigator grant (INVES19041ALVA) and currently receives "Ayudas Ramon y Cajal" (RYC2021-033381) from the MCIN/AEI/10.13039/501100011033. F.A. receives a Miguel Servet I (CP19/00114) contract from ISCIII (Instituto de Salud Carlos III) co-financed by FSE (Fondo Social Europeo) "Investing in Your Future." I.M. receives support from the Ministry of Economy and Competitiveness and Spanish Ministry of Research (MINECO SAF2014-52361-R and SAF 2017-83267-C2-1R and PID2020-112892RB-100, PID2020-113174-RA-100 [AEI/FEDER,UE]), Cancer Research Institute under the CRI-CLIP, Asociación Española Contra el Cancer (AECC) Foundation under grant GCB15152947MELE, Joint Translational Call for Proposals 2015 (JTC 2015) TRANSCAN-2 (code: TRS-2016-00000371 M.A. is supported by an AECC grant (INVES19041ALVA). A.T. received financial support through Spanish Ministry of Science (RyC 2019-026406-I).

AUTHOR CONTRIBUTIONS

Conceptualization, C.A.D.T., P.B., and I.M.; Formal Analysis, C.A.D.T. and P.B.; Funding Acquisition, P.B. and I.M.; Investigation, C.A.D.T., A.C., L.A., M.A., Á.B., M.F.-S., J.S.R.-C., I.O., C.G., N.A., E.B., A.T., J.G.-G., F.A., A.S., S.H., B.P., S.M.J.v.D., and A.v.E.; Methodology, C.A.D.T., A.C., E.B., P.B., and I.M.; Project Administration, B.P.; Supervision, P.B., I.M., I.O., A.T., M.A., A.v.E., and S.M.J.v.D.; Visualization, C.A.D.T. and L.A.; Writing – Original Draft, C.A.D.T. and P.B.; Writing – Review & Editing, C.A.D.T., P.B., I.M., A.C., L.A., M.A., Á.B., M.F.-S., J.S.R.-C., I.O., C.G., N.A., E.B., A.T., J.G.-G., B.P., F.A., A.S., S.H., S.M.J.v.D., and A.v.E.

DECLARATION OF INTERESTS

I.M. reports advisory roles with Roche-Genentech, Bristol Myers Squibb, CYTOMX, Incyte, MedImmune, Tusk, F-Star, Genmab, Molecular Partners, Alligator, Bioncotech, Merck Sharpe & Dohme, Merck Serono, Boehringer Ingelheim, AstraZeneca, Numab, Catalym, and Bayer and research funding from Roche, Bristol Myers Squibb, Alligator, and Highlight Therapeutics.

REFERENCES

1. Berraondo, P., Sanmamed, M.F., Ochoa, M.C., Etxeberria, I., Aznar, M.A., Pérez-Gracia, J.L., Rodríguez-Ruiz, M.E., Ponz-Sarvise, M., Castañón, E., and Melero, I. (2019). Cytokines in clinical cancer immunotherapy. Br. J. Cancer *120*, 6–15.
2. Guedan, S., Calderon, H., Posey, A.D., Jr., and Maus, M.V. (2019). Emerging Cellular Therapies for Cancer. Annu. Rev. Immunol. *12*, 145–156.
3. Ribas, A., and Wolchok, J.D. (2018). Cancer immunotherapy using checkpoint blockade. Science *359*, 1350–1355.
4. Upadhaya, S., Neftelinov, S.T., Hodge, J., and Campbell, J. (2022). Challenges and opportunities in the PD1/PDL1 inhibitor clinical trial landscape. Nat. Rev. Drug Discov. *21*, 482–483.
5. Sharma, P., Hu-Lieskovan, S., Wargo, J.A., and Ribas, A. (2017). Primary, Adaptive, and Acquired Resistance to Cancer Immunotherapy. Cell *168*, 707–723.
6. Sanmamed, M.F., Berraondo, P., Rodriguez-Ruiz, M.E., and Melero, I. (2022). Charting roadmaps towards novel and safe synergistic immunotherapy combinations. Nat. Cancer *3*, 665–680.
7. Tugues, S., Burkhard, S.H., Ohs, I., Vrohlings, M., Nussbaum, K., Vom Berg, J., Kulig, P., and Becher, B. (2015). New insights into IL-12-mediated tumor suppression. Cell Death Differ. *22*, 237–246.
8. Lund, R.J., Chen, Z., Scheinin, J., and Lahesmaa, R. (2004). Early target genes of IL-12 and STAT4 signaling in th cells. J. Immunol. *172*, 6775–6782.
9. Morinobu, A., Gadina, M., Strober, W., Visconti, R., Fornace, A., Montagna, C., Feldman, G.M., Nishikomori, R., and O'Shea, J.J. (2002). STAT4 serine phosphorylation is critical for IL-12-induced IFN-gamma production but not for cell proliferation. Proc. Natl. Acad. Sci. USA *99*, 12281–12286.
10. Sgadari, C., Angiolillo, A.L., and Tosato, G. (1996). Inhibition of angiogenesis by interleukin-12 is mediated by the interferon-inducible protein 10. Blood *87*, 3877–3882.
11. Kerkar, S.P., Goldszmid, R.S., Muranski, P., Chinnasamy, D., Yu, Z., Reger, R.N., Leonardi, A.J., Morgan, R.A., Wang, E., Marincola, F.M., et al. (2011). IL-12 triggers a programmatic change in dysfunctional myeloid-derived cells within mouse tumors. J. Clin. Invest. *121*, 4746–4757.
12. Rosa, F.M., and Fellous, M. (1988). Regulation of HLA-DR gene by IFN-gamma. Transcriptional and post-transcriptional control. J. Immunol. *140*, 1660–1664.
13. Taub, D.D., Lloyd, A.R., Conlon, K., Wang, J.M., Ortaldo, J.R., Harada, A., Matsushima, K., Kelvin, D.J., and Oppenheim, J.J. (1993). Recombinant human interferon-inducible protein 10 is a chemoattractant for human monocytes and T lymphocytes and promotes T cell adhesion to endothelial cells. J. Exp. Med. *177*, 1809–1814.
14. Atkins, M.B., Robertson, M.J., Gordon, M., Lotze, M.T., DeCoste, M., DuBois, J.S., Ritz, J., Sandler, A.B., Edington, H.D., Garzone, P.D., et al. (1997). Phase I evaluation of intravenous recombinant human interleukin 12 in patients with advanced malignancies. Clin. Cancer Res. *3*, 409–417.
15. Leonard, J.P., Sherman, M.L., Fisher, G.L., Buchanan, L.J., Larsen, G., Atkins, M.B., Sosman, J.A., Dutcher, J.P., Vogelzang, N.J., and Ryan, J.L. (1997). Effects of single-dose interleukin-12 exposure on interleukin-12-associated toxicity and interferon-gamma production. Blood *90*, 2541–2548.
16. Cirella, A., Luri-Rey, C., Di Trani, C.A., Teijeira, A., Olivera, I., Bolaños, E., Castañón, E., Palencia, B., Brocco, D., Fernández-Sendin, M., et al. (2022). Novel strategies exploiting interleukin-12 in cancer immunotherapy. Pharmacol. Ther. *239*, 108189.
17. Hewitt, S.L., Bailey, D., Zielinski, J., Apte, A., Musenge, F., Karp, R., Burke, S., Garcon, F., Mishra, A., Gurumurthy, S., et al. (2020). Intratumoral IL12 mRNA Therapy Promotes TH1 Transformation of the Tumor Microenvironment. Clin. Cancer Res. *26*, 6284–6298.
18. Hewitt, S.L., Bai, A., Bailey, D., Ichikawa, K., Zielinski, J., Karp, R., Apte, A., Arnold, K., Zacharek, S.J., Iliou, M.S., et al. (2019). Durable anticancer immunity from intratumoral administration of IL-23, IL-36γ, and OX40L mRNAs. Sci. Transl. Med. *11*, eaat9143.
19. Hotz, C., Wagenaar, T.R., Gieseke, F., Bangari, D.S., Callahan, M., Cao, H., Diekmann, J., Diken, M., Grunwitz, C., Hebert, A., et al. (2021). Local delivery of mRNA-encoded cytokines promotes antitumor immunity and tumor eradication across multiple preclinical tumor models. Sci. Transl. Med. *13*, eabc7804.

20. Cirella, A., Bolanos, E., Di Trani, C.A., de Andrea, C.E., Sánchez-Gregorio, S., Etxeberria, I., Gonzalez-Gomariz, J., Olivera, I., Brocco, D., Glez-Vaz, J., et al. (2022). Intratumoral Gene Transfer of mRNAs Encoding Interleukin-12 in Combination with Decoy-Resistant Interleukin-18 Improves Local and Systemic Antitumor Immunity (Cancer Immunol Res).

21. Di Trani, C.A., Fernandez-Sendin, M., Cirella, A., Segués, A., Olivera, I., Bolaños, E., Melero, I., and Berraondo, P. (2022). Advances in mRNA-based drug discovery in cancer immunotherapy. Expert Opin. Drug Discov. *17*, 41–53.

22. Melero, I., Castanon, E., Alvarez, M., Champiat, S., and Marabelle, A. (2021). Intratumoural administration and tumour tissue targeting of cancer immunotherapies. Nat. Rev. Clin. Oncol. *18*, 558–576.

23. Marabelle, A., Tselikas, L., de Baere, T., and Houot, R. (2017). Intratumoral immunotherapy: using the tumor as the remedy. Ann. Oncol. *28*, xii33–xii43.

24. Di Trani, C.A., Cirella, A., Arrizabalaga, L., Fernandez-Sendin, M., Bella, A., Aranda, F., Melero, I., and Berraondo, P. (2022). Overcoming the limitations of cytokines to improve cancer therapy. Int. Rev. Cell Mol. Biol. *369*, 107–141.

25. Meininger, L., Madan, R.A., Toney, N., Donahue, R.N., Gatti-Mays, M.E., Strauss, J., Karzai, F., Bilusic, M., Marte, J.L., Cordes, L.M., et al. (2022). The immunocytokine M9241 in the treatment of prostate cancer (PCa): Clinical and immune data from a phase 1 study. J. Clin. Oncol. *40*, 127.

26. Lieschke, G.J., Rao, P.K., Gately, M.K., and Mulligan, R.C. (1997). Bioactive murine and human interleukin-12 fusion proteins which retain antitumor activity *in vivo*. Nat. Biotechnol. *15*, 35–40.

27. Van der Jeught, K., Joe, P.T., Bialkowski, L., Heirman, C., Daszkiewicz, L., Liechtenstein, T., Escors, D., Thielemans, K., and Breckpot, K. (2014). Intratumoral administration of mRNA encoding a fusokine consisting of IFN-β and the ectodomain of the TGF-β receptor II potentiates antitumor immunity. Oncotarget *5*, 10100–10113.

28. Probst, J., Weide, B., Scheel, B., Pichler, B.J., Hoerr, I., Rammensee, H.G., and Pascolo, S. (2007). Spontaneous cellular uptake of exogenous messenger RNA *in vivo* is nucleic acid-specific, saturable and ion dependent. Gene Ther. *14*, 1175–1180.

29. Van Lint, S., Renmans, D., Broos, K., Goethals, L., Maenhout, S., Benteyn, D., Goyvaerts, C., Du Four, S., Van der Jeught, K., Bialkowski, L., et al. (2016). Intratumoral Delivery of TriMix mRNA Results in T-cell Activation by Cross-Presenting Dendritic Cells. Cancer Immunol. Res. *4*, 146–156.

30. Segal, J.G., Lee, N.C., Tsung, Y.L., Norton, J.A., and Tsung, K. (2002). The role of IFN-gamma in rejection of established tumors by IL-12 : source of production and target. Cancer Res. *62*, 4696–4703.

31. Brunda, M.J., Luistro, L., Hendrzak, J.A., Fountoulakis, M., Garotta, G., and Gately, M.K. (1995). Role of interferon-gamma in mediating the antitumor efficacy of interleukin-12. J. Immunother. Emphasis Tumor Immunol. *17*, 71–77.

32. Carson, W.E., Dierksheide, J.E., Jabbour, S., Anghelina, M., Bouchard, P., Ku, G., Yu, H., Baumann, H., Shah, M.H., Cooper, M.A., et al. (2000). Coadministration of interleukin-18 and interleukin-12 induces a fatal inflammatory response in mice: critical role of natural killer cell interferon-gamma production and STAT-mediated signal transduction. Blood *96*, 1465–1473.

33. Cirella, A., Berraondo, P., Di Trani, C.A., and Melero, I. (2020). Interleukin-12 Message in a Bottle. Clin. Cancer Res. *26*, 6080–6082.

34. Stanley, E.R., and Chitu, V. (2014). CSF-1 receptor signaling in myeloid cells. Cold Spring Harb. Perspect. Biol. *6*, a021857.

35. Kirma, N., Hammes, L.S., Liu, Y.G., Nair, H.B., Valente, P.T., Kumar, S., Flowers, L.C., and Tekmal, R.R. (2007). Elevated expression of the oncogene c-fms and its ligand, the macrophage colony-stimulating factor-1, in cervical cancer and the role of transforming growth factor-beta1 in inducing c-fms expression. Cancer Res. *67*, 1918–1926.

36. Neubert, N.J., Schmittnaegel, M., Bordry, N., Nassiri, S., Wald, N., Martignier, C., Tillé, L., Homicsko, K., Damsky, W., Maby-El Hajjami, H., et al. (2018). T cell-induced CSF1 promotes melanoma resistance to PD1 blockade. Sci. Transl. Med. *10*, eaan3311.

37. Zhu, Y., Knolhoff, B.L., Meyer, M.A., Nywening, T.M., West, B.L., Luo, J., Wang-Gillam, A., Goedegebuure, S.P., Linehan, D.C., and DeNardo, D.G. (2014). CSF1/CSF1R blockade reprograms tumor-infiltrating macrophages and improves response to T-cell checkpoint immunotherapy in pancreatic cancer models. Cancer Res. *74*, 5057–5069.

38. Cannarile, M.A., Weisser, M., Jacob, W., Jegg, A.M., Ries, C.H., and Rüttinger, D. (2017). Colony-stimulating factor 1 receptor (CSF1R) inhibitors in cancer therapy. J. Immunother. Cancer *5*, 53.

39. Dong, H., Strome, S.E., Salomao, D.R., Tamura, H., Hirano, F., Flies, D.B., Roche, P.C., Lu, J., Zhu, G., Tamada, K., et al. (2002). Tumor-associated B7-H1 promotes T-cell apoptosis: a potential mechanism of immune evasion. Nat. Med. *8*, 793–800.

40. Quetglas, J.I., Labiano, S., Aznar, M.Á., Bolaños, E., Azpilikueta, A., Rodriguez, I., Casales, E., Sánchez-Paulete, A.R., Segura, V., Smerdou, C., and Melero, I. (2015). Virotherapy with a Semliki Forest Virus-Based Vector Encoding IL12 Synergizes with PD-1/PD-L1 Blockade. Cancer Immunol. Res. *3*, 449–454.

41. Silva-Pilipich, N., Lasarte-Cía, A., Lozano, T., Martín-Otal, C., Lasarte, J.J., and Smerdou, C. (2022). Intratumoral electroporation of a self-amplifying RNA expressing IL-12 induces antitumor effects in mouse models of cancer. Mol. Ther. Nucleic Acids *29*, 387–399.

42. Sahin, U., Karikó, K., and Türeci, Ö. (2014). mRNA-based therapeutics–developing a new class of drugs. Nat. Rev. Drug Discov. *13*, 759–780.

43. Rohner, E., Yang, R., Foo, K.S., Goedel, A., and Chien, K.R. (2022). Unlocking the promise of mRNA therapeutics. Nat. Biotechnol. *40*, 1586–1600.

44. Taina-González, L., and de la Fuente, M. (2022). The Potential of Nanomedicine to Unlock the Limitless Applications of mRNA. Pharmaceutics *14*, 460.

45. Cheng, Q., Wei, T., Farbiak, L., Johnson, L.T., Dilliard, S.A., and Siegwart, D.J. (2020). Selective organ targeting (SORT) nanoparticles for tissue-specific mRNA delivery and CRISPR-Cas gene editing. Nat. Nanotechnol. *15*, 313–320.

46. Etxeberria, I., Bolaños, E., Quetglas, J.I., Gros, A., Villanueva, A., Palomero, J., Sánchez-Paulete, A.R., Piulats, J.M., Matias-Guiu, X., Olivera, I., et al. (2019). Intratumor Adoptive Transfer of IL-12 mRNA Transiently Engineered Antitumor CD8. Cancer Cell *36*, 613–629.e7.

47. Di Trani, C.A., Cirella, A., Arrizabalaga, L., Bella, Á., Fernandez-Sendin, M., Russo-Cabrera, J.S., Gomar, C., Olivera, I., Bolaños, E., González-Gomariz, J., et al. (2023). Intracavitary adoptive transfer of IL-12 mRNA-engineered tumor-specific CD8. OncoImmunology *12*, 2147317.

48. Holtkamp, S., Kreiter, S., Selmi, A., Simon, P., Koslowski, M., Huber, C., Türeci, O., and Sahin, U. (2006). Modification of antigen-encoding RNA increases stability, translational efficacy, and T-cell stimulatory capacity of dendritic cells. Blood *108*, 4009–4017.

49. Jerby-Arnon, L., Shah, P., Cuoco, M.S., Rodman, C., Su, M.J., Melms, J.C., Leeson, R., Kanodia, A., Mei, S., Lin, J.R., et al. (2018). A Cancer Cell Program Promotes T Cell Exclusion and Resistance to Checkpoint Blockade. Cell *175*, 984–997.e24.

50. Lee, H.O., Hong, Y., Etlioglu, H.E., Cho, Y.B., Pomella, V., Van den Bosch, B., Vanhecke, J., Verbandt, S., Hong, H., Min, J.W., et al. (2020). Lineage-dependent gene expression programs influence the immune landscape of colorectal cancer. Nat. Genet. *52*, 594–603.

51. Amezquita, R.A., Lun, A.T.L., Becht, E., Carey, V.J., Carpp, L.N., Geistlinger, L., Marini, F., Rue-Albrecht, K., Risso, D., Soneson, C., et al. (2020). Orchestrating single-cell analysis with Bioconductor. Nat. Methods *17*, 137–145.

52. Hao, Y., Hao, S., Andersen-Nissen, E., Mauck, W.M., Zheng, S., Butler, A., Lee, M.J., Wilk, A.J., Darby, C., Zager, M., et al. (2021). Integrated analysis of multimodal single-cell data. Cell *184*, 3573–3587.e29.

OMTN, Volume *33*

Supplemental information

Intratumoral injection of IL-12-encoding mRNA targeted to CSFR1 and PD-L1 exerts potent anti-tumor effects without substantial systemic exposure

Claudia Augusta Di Trani, Assunta Cirella, Leire Arrizabalaga, Maite Alvarez, Ángela Bella, Myriam Fernandez-Sendin, Joan Salvador Russo-Cabrera, Celia Gomar, Nuria Ardaiz, Alvaro Teijeira, Elixabet Bolaños, José González-Gomariz, Itziar Otano, Fernando Aranda, Belén Palencia, Aina Segués, Shuyu Huang, Sander M.J. van Duijnhoven, Andrea van Elsas, Ignacio Melero, and Pedro Berraondo

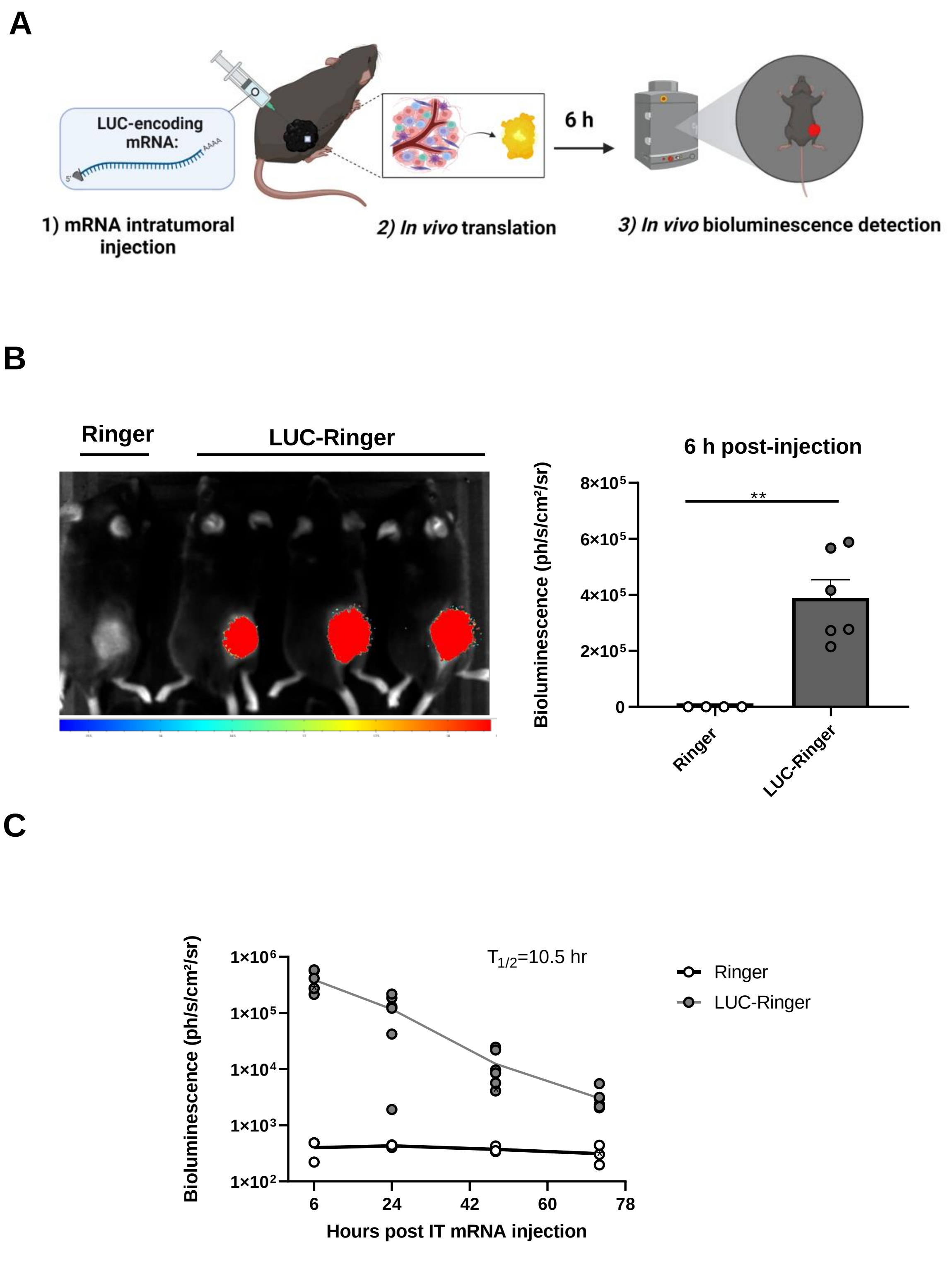

Figure S1. Intratumoral naked luciferase mRNA delivery leads to high expression in the tumor niche. A. Schematic representation of the experimental procedure. B16-OVA cells (0.5 x 10^6) were inoculated subcutaneously into the right flank of C57BL/6 mice (n=4-

6). Twelve days post-tumor inoculation, 10 µg of mRNA encoding luciferase were delivered intratumorally in 50 µl of Ringer's lactate solution. **B.** *In vivo* bioluminescence measured 6 h post-mRNA injection. Representative mice from each group are shown. The graph on the right includes the quantification of all mice in each experimental group. **C.** Bioluminescence follow-up. Expression half-life was determined by fitting it to a one-phase decay equation. Data are given as mean ± SEM, and statistical significance was determined with two-tailed unpaired t-test (Mann-Whitney) for panel B (**$p < 0.01$).

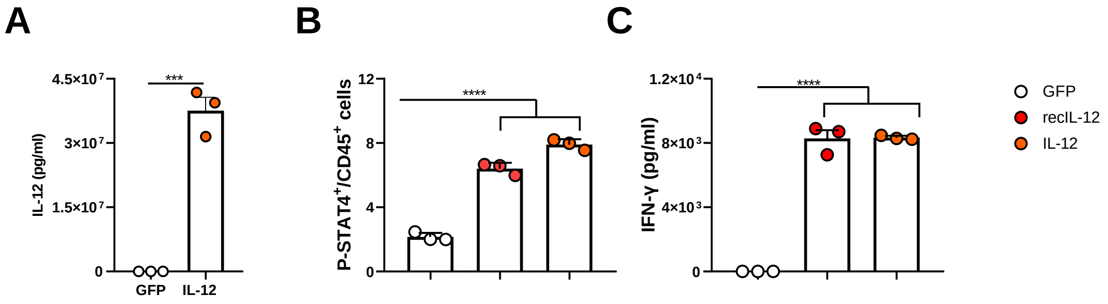

Figure S2. The mRNA-encoded scIL-12 is correctly secreted and bioactive *in vitro*. A. B16-OVA cells were transfected with mRNA encoding GFP or IL-12 formulated with the TransIT-mRNA Transfection kit. Supernatants were collected 72 h post-transfection and assayed for IL-12p70 detection by ELISA. **B.** Murine splenocytes were preactivated O/N using plate-bound anti-CD3 (1 µg/ml) and soluble anti-CD28 (1 µg/ml). The day after, 3 x 10^6 preactivated splenocytes were incubated for 1 h at 37 °C with the conditioned media from mRNA-transfected B16-OVA cells, containing 400 ng of IL-12, as previously assessed by the IL-12p70 ELISA sandwich assay. As a positive control, 400 ng of recombinant IL-12 were used and the supernatant of cells transfected with GFP-mRNA was employed as a negative control. Then, cells were washed and the pellet was resuspended in Cytofix buffer for 15 mins at 37 °C. After extensive washing, cells were permeabilized using Perm Buffer III for 30 mins at 4 °C and then stained with anti-CD45-PerCP and anti-P-STAT4-PE antibody for 45 minutes at RT. The percentage of $CD45^+$ cells with phosphorylated STAT-4 are shown. **C.** Murine splenocytes were preactivated in an anti-CD3-coated plate O/N. Then, they were treated with the supernatants collected 72 h after mRNA transfection of B16-OVA cells or with 50 ng of recombinant IL-12 as a positive control. Forty-eight hours after incubation, the splenocyte culture supernatants were analyzed for IFN-γ production by ELISA. Data are shown as mean ± SEM. Statistical significance was determined with one-way ANOVA followed by Dunnett's multiple comparison tests (***$p < 0.001$, ****$p < 0.0001$).

A

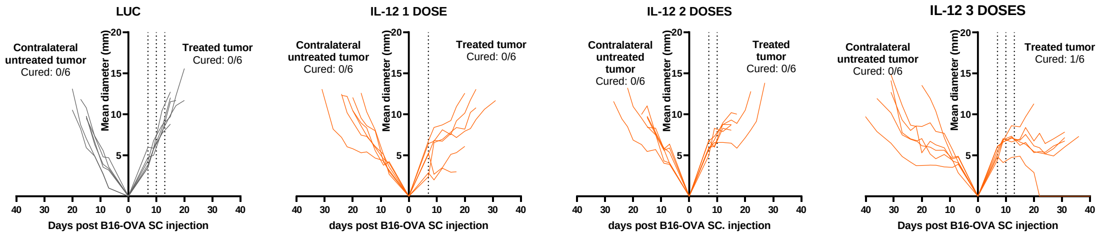

B

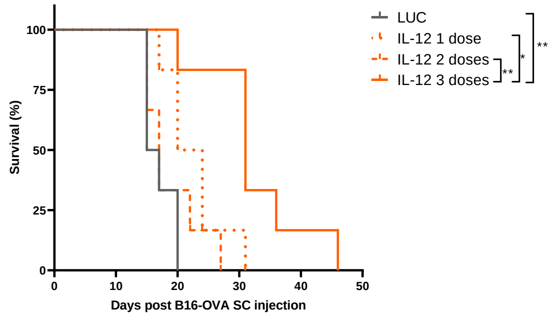

Figure S3. Three injections of IL-12-mRNA outperform a single or two doses in terms of antitumor efficacy. A. C57BL/6 mice (n=6) were injected with 0.5 x 10^6 B16-OVA cells subcutaneously in the right flank and 0.15 x 10^6 in the left flank. At the indicated time (dashed lines), mRNAs encoding LUC and IL-12 (0.5 µg/dose) were delivered intratumorally in 50 µl of Ringer's lactate solution. The one dose group was treated at day 7. The 2 dose-group was treated at day 7 and 10, and the 3 dose-groups (LUC and IL-12) were treated at day 7, 10 and 13. Individual follow-up of tumor sizes with the fraction of cured tumors is presented. **B.** Survival follow-up is shown and the data were analyzed by Log-rank (Mantel-Cox) tests (*$p < 0.05$, **$p < 0.01$).

A

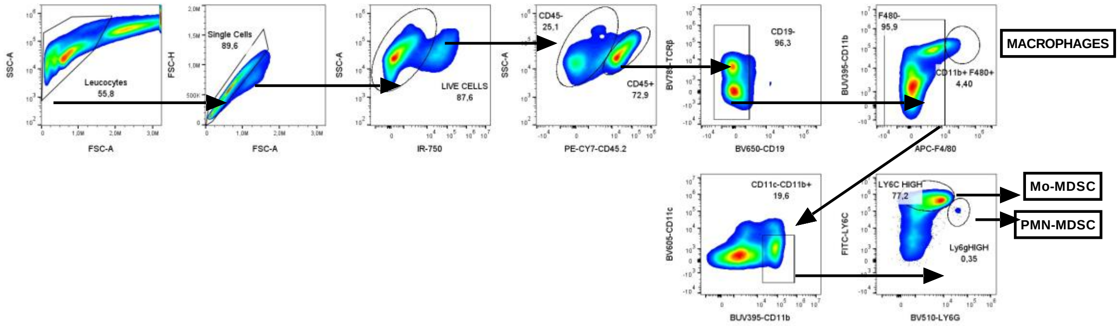

B

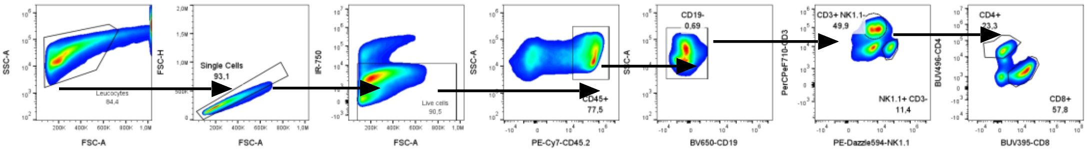

Figure S4. Gating strategy of multi-color flow cytometry analyses. A. Gating strategy employed in flow cytometry analyses to classify myeloid cell populations in Figs. 3-4-7. **B.** Gating strategy employed in flow cytometry analyses to classify lymphoid cell populations in Fig. 4 and Fig. S6.

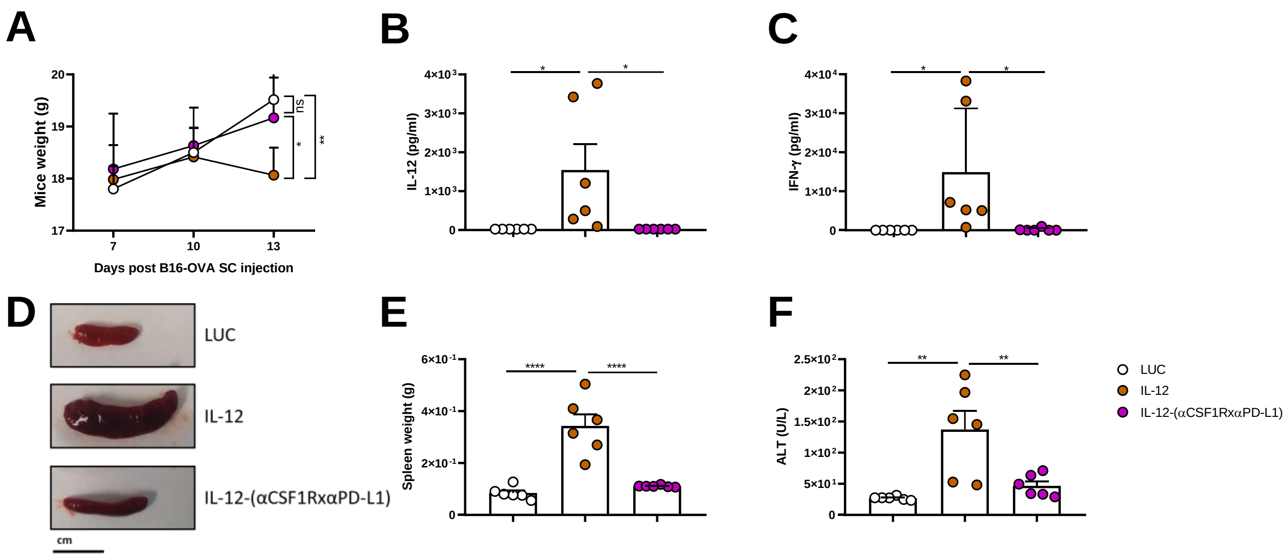

Figure S5. Mice treated with the mRNA encoding the IL-12-diabody are protected from systemic toxicity observed following the intratumoral administration of non-chimerized IL-12-mRNA. C57BL/6 mice were injected with 0.5 x 10^6 B16-OVA cells subcutaneously in both flanks (n=6/group). On days 7, 10, and 13 after tumor inoculation, each implanted tumor was injected with 10 µg of each indicated mRNA. **A.** Total body weight (g) measured before each dose injection. Statistical comparison of data collected on day 13. **B-F.** Twenty-four hours after the third dose, serum was collected from the mice for subsequent analyses, and mice were sacrificed. **B.** IL-12p70 and **C.** IFN-γ systemic levels were assessed in mice sera by commercial ELISA assays. **D.** Representative image of spleens for each condition. **E.** Quantification of spleen weights. **F.** Alanine transaminase in sera of mice measured with an automatic biochemical analyzer Cobas c-311 (Roche Diagnostics). Data are shown as mean ± SEM. Statistical significance was determined with one-way ANOVA followed by Tukey's multiple comparison tests in panels A, B, C, E, F (*p < 0.05, **p < 0.01, ****p < 0.0001).

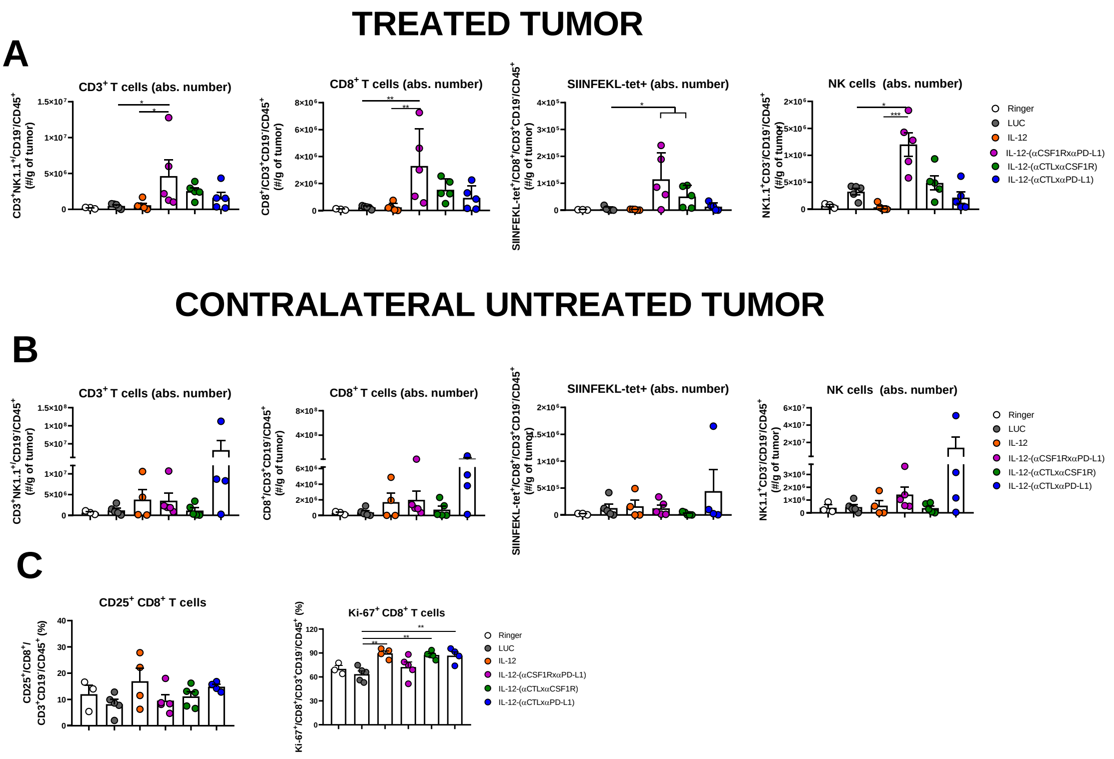

Figure S6. Lymphocyte infiltration in treated and uninjected lesions. A. B16-OVA-bearing mice (n=3-5) received two doses of the indicated mRNAs on day 7 and day 10 after tumor injection and were sacrificed 4 days after the last treatment (0.5 µg of IL-12-encoding mRNA and 10 µg of the mRNAs encoding the diabodies were used per each dose). Immune cells of the excised tumors were phenotypically characterized by multi-color flow cytometry analysis. **A.** Absolute numbers of different immune populations in treated tumors and **B.** contralateral untreated tumors are shown. **C.** The activation and proliferation states of $CD8^+$ T cells in contralateral tumors were evaluated. Data are shown as mean ± SEM. Statistical significance was determined with one-way ANOVA followed by Sidak's multiple comparisons tests (*p<0.05, **p<0.001, ***p < 0.001).

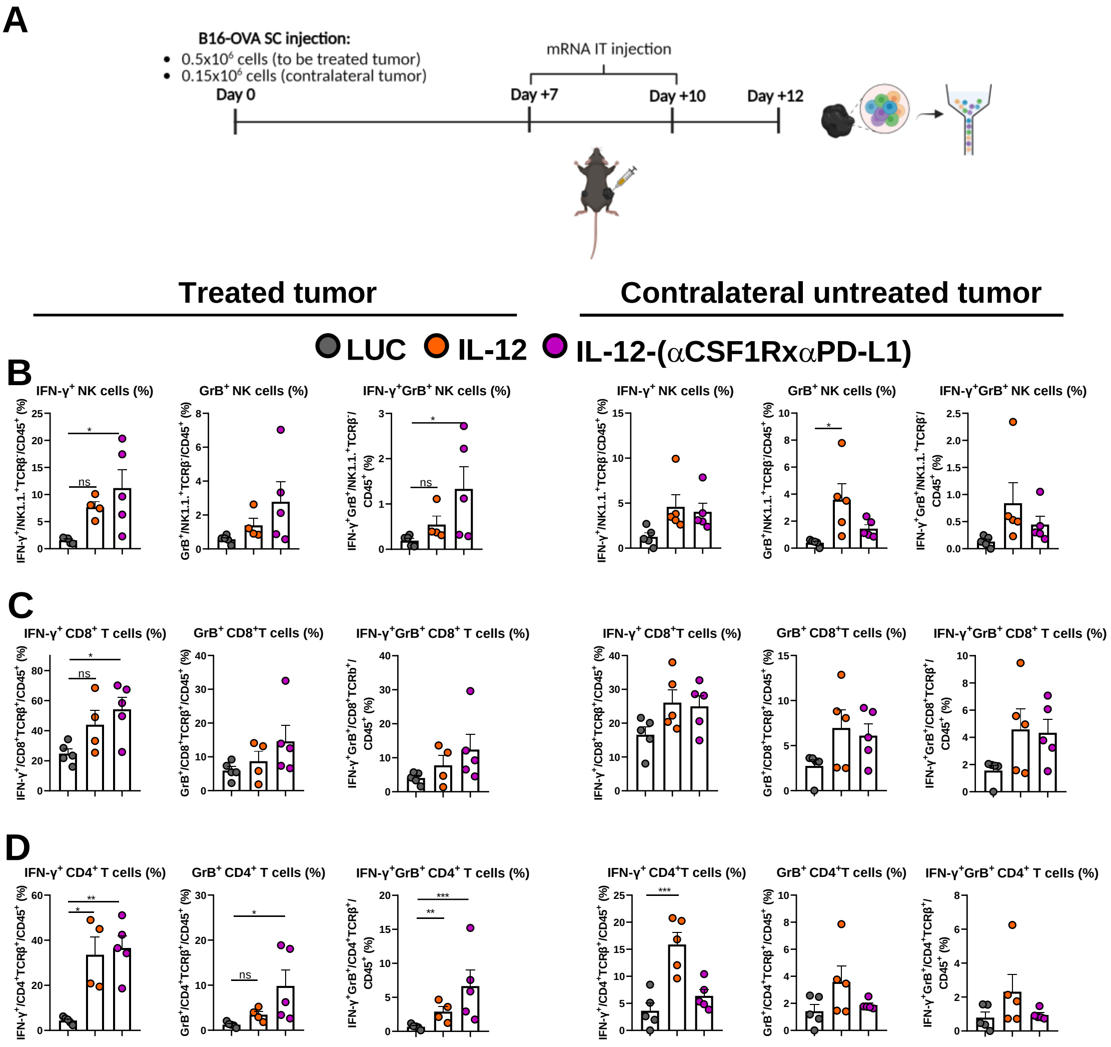

Figure S7. Expression of IFN-γ and granzyme B in T lymphocytes and NK cells infiltrating the treated and contralateral untreated tumors. A. Scheme summarizing the treatment regimen and the experimental timeline including the flow cytometry analysis. B16-OVA-bearing mice (n=5) received two doses of the indicated mRNAs on day 7 and day 10 after tumor injection and were sacrificed two days after the last treatment. 0.5 µg of IL-12-encoding mRNA and 10 µg of the mRNA encoding IL-12-(αCSF1RxαPD-L1) and LUC were used. Immune cells of the excised tumors were characterized by multi-color flow cytometry analysis. Data are shown as mean ± SEM. Statistical significance was

determined with one-way ANOVA followed by Tukey's multiple comparisons (*$p < 0.05$, **$p < 0.01$).

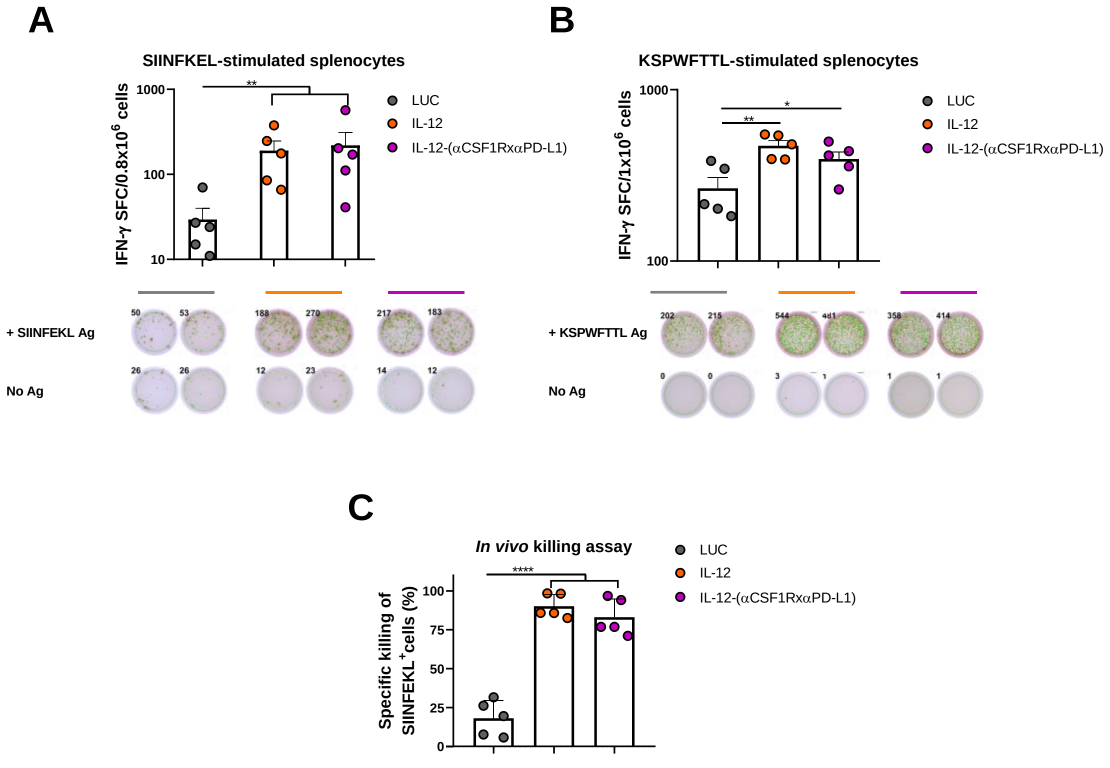

Figure S8. Targeted and untargeted IL-12 trigger a systemic antitumor immune response. A. C57BL/6 mice were injected with 0.5 x 10^6 B16-OVA cells subcutaneously in the right flank and 0.15 x 10^6 in the left flank (n=5/group). Mice received two injections of 10 µg of LUC-mRNA, 0.5 µg of IL-12-mRNA, 10 µg of IL-12-(αCSF1RxαPD-L1)-mRNA (on day 7 and 10 after tumor inoculation). Forty-eight hours after the second mRNA dose, 0.8 x 10^6 splenocytes were stimulated with 0.001 mg/ml $OVA_{257\text{-}264}$ peptide or with medium alone and incubated on an IFN-γ ELISpot plate. After 24 h, the spot-forming units were counted with an automated ImmunoSpot counter. Results correspond to data after subtraction of values obtained in control wells without peptide. Below the graph, two representative mice of each condition are shown. **B.** C57BL/6 mice were injected with 0.5 x 10^6 MC38 cells subcutaneously in the right flank and 0.2 x 10^6 in the left flank (n=5/group). Mice received two inoculations of 10 µg of LUC-mRNA, 0.5 µg of IL-12-mRNA, 10 µg of IL-12-(αCSF1RxαPD-L1)-mRNA (on day 6 and 9 after tumor inoculation). Forty-eight hours after the second mRNA dose, 1 x 10^6 splenocytes were

stimulated with 0.01 mg/ml KSPWFTTL peptide or with medium alone and incubated on an IFN-γ ELISpot plate. After 24 h, the spot-forming units were counted with an automated ImmunoSpot counter. Results correspond to data after subtraction of values obtained in control wells without peptide. Below each graph, two representative mice of each condition are shown. **C.** C57BL/6 mice were injected with 0.5 x 10^6 B16-OVA cells subcutaneously in the right flank and 0.15 x 10^6 in the left flank (n=5/group). Mice received three inoculations of 10 μg of LUC-mRNA, 0.5 μg of IL-12-mRNA, 10 μg of IL-12-(αCSF1RxαPD-L1)-mRNA (on day 7, 10 and 13 after tumor inoculation). In order to measure *in vivo* killing capability of $CD8^+$ T cells, single-cell suspensions of splenocytes were obtained from naive mice. Half of the splenocytes were pulsed for 30 mins at 37 ºC with 0.01 mg/ml $OVA_{257\text{-}264}$ peptide and the other half was incubated with the same volume of PBS only. After extensive washing, the two populations were stained with 5mM and 0.5mM of CFSE dye, respectively, and were mixed in a 1:1 ratio in order to retro-orbitally inject 10 x 10^6 cells per mouse 24 h after the third mRNA dose. 20 h post-transfer of the cells, spleens were isolated, processed and single-cell suspensions were analyzed by flow cytometry. To calculate the percentage of specific lysis of the target cell population, the following equation was used for each treated mouse, compared to naïve control: %specific lysis = 100 - [100*(%$CFSE^{high}$ immunized mouse/% $CFSE^{low}$ immunized mouse)/(% $CFSE^{high}$ naïve mouse/% $CFSE^{low}$ naïve mouse). Data are shown as mean ± SEM. Statistical significance was determined with one-way ANOVA followed by Dunnett's multiple comparison tests of Log-transformed data for panels A and B and followed by Tukey's multiple comparisons for panel C (*$p < 0.05$, **$p < 0.01$, ****$p < 0.0001$).

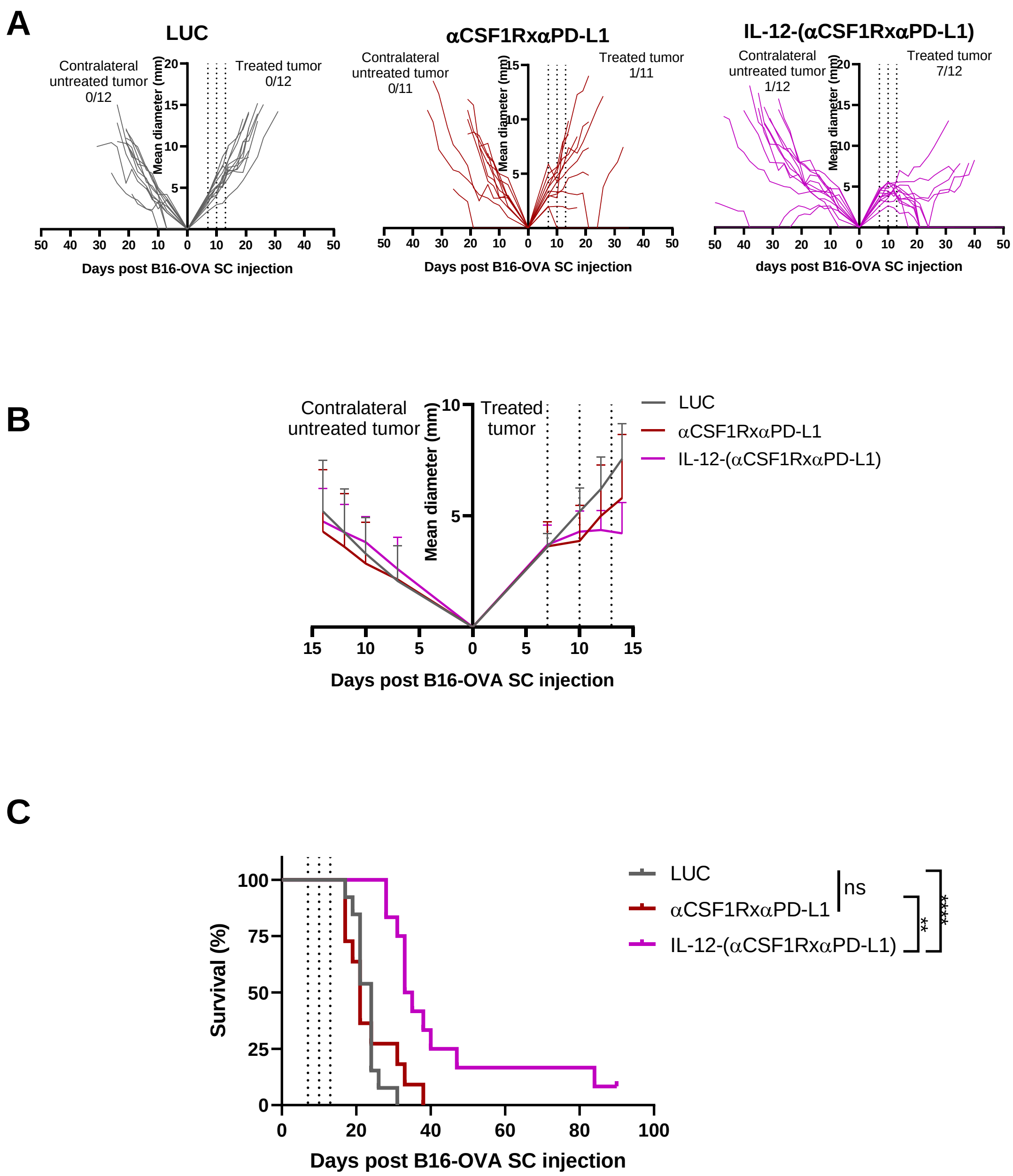

Figure S9. IL-12 is essential for the antitumor efficacy of the diabody-encoded mRNAs. Mice were injected with the B16-OVA cell line in both flanks and treated with the 10 µg of the indicated constructs on day 7, 10, and 13 after tumor cell inoculation. Two independent experiments were pooled (n=11-12). **A.** Individual follow-up of tumor sizes where the fraction of cured tumors is presented. **B.** Cumulative data of panel B. **C.** Mice survival follow-up of 2 pooled experiments is shown. Survival data in panel C were analyzed by Log-rank (Mantel-Cox) tests.

A

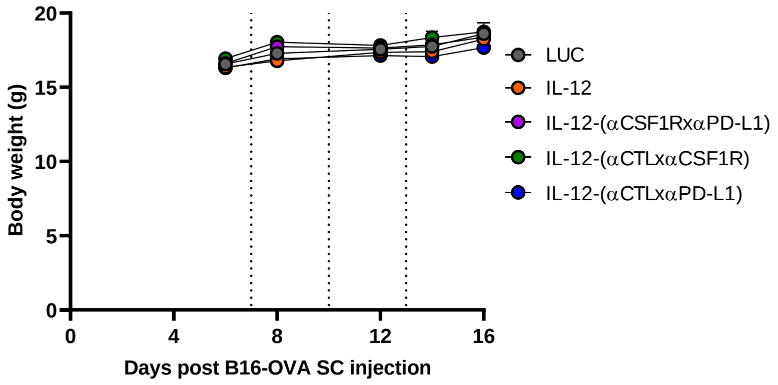

B

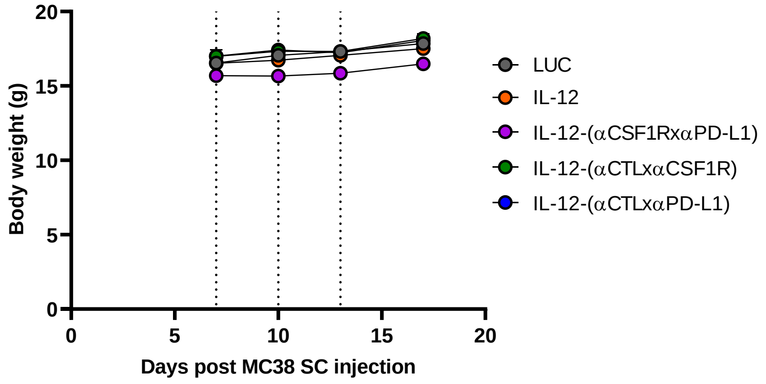

Figure S10. Treatments with mRNA-encoding IL-12-carrying constructs at the therapeutic dose do not induce toxicity in terms of weight loss. A. Mice of the antitumor experiments shown in Fig. 5B were weighted throughout the experiment starting from one day before the first mRNA injection. **B.** Mice of the antitumor experiments shown in Fig. 6B were weighed throughout the experiment, starting from just before the first mRNA injection. Statistical significance was determined with one-way ANOVA followed by Sidak's multiple comparisons tests. No significant differences were detected.

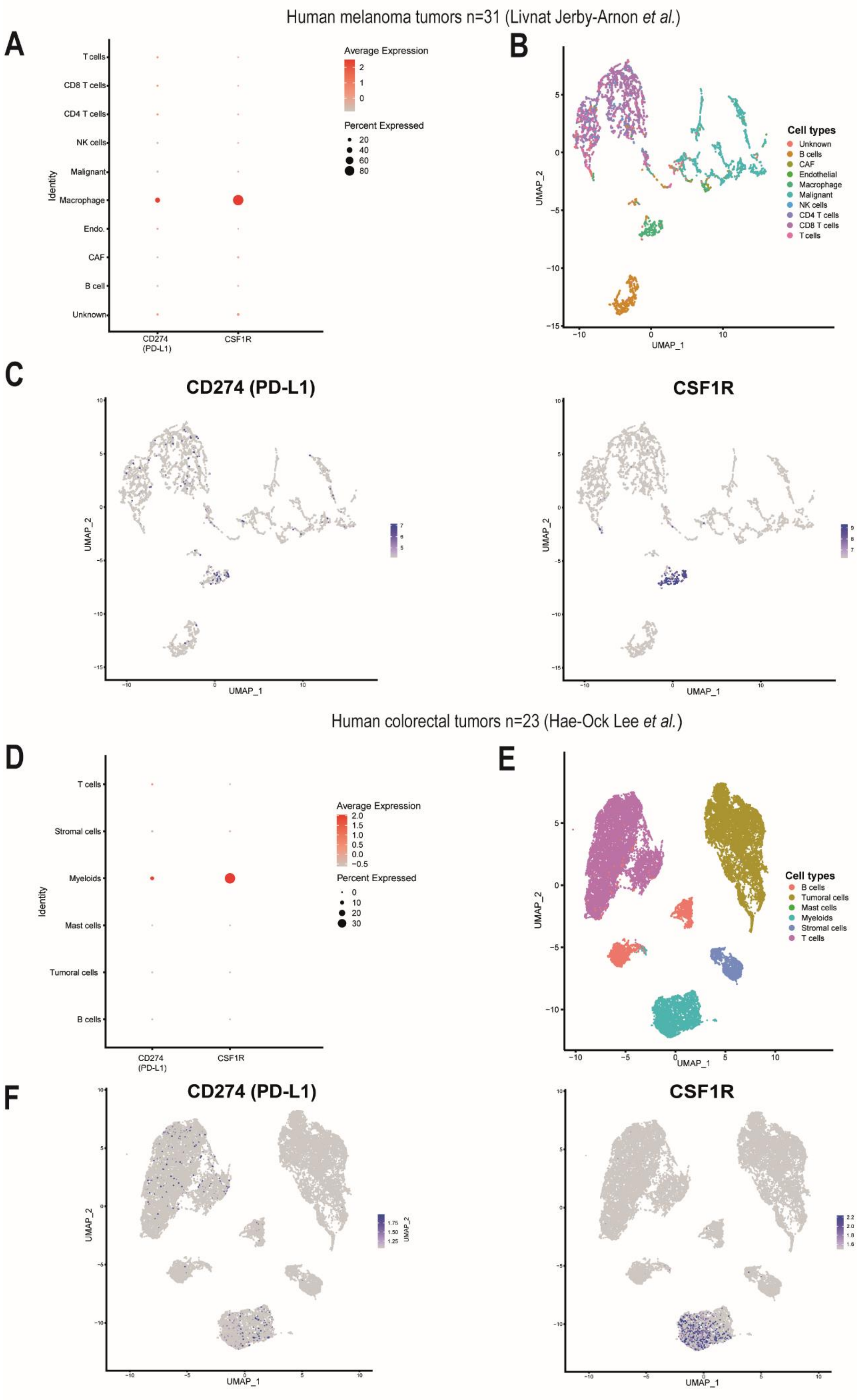
Human melanoma tumors n=31 (Livnat Jerby-Arnon et al.)
A
B
T cells
CD8 T cells
CD4 T cells
NK cells
Malignant
Macrophage
Endo.
CAF
B cell
Unknown
Identity
CD274 (PD-L1)
CSF1R
Average Expression
Percent Expressed
Cell types
Unknown
B cells
CAF
Endothelial
Macrophage
Malignant
NK cells
CD4 T cells
CD8 T cells
T cells
UMAP_1
UMAP_2
C
CD274 (PD-L1)
CSF1R
Human colorectal tumors n=23 (Hae-Ock Lee et al.)
D
E
T cells
Stromal cells
Myeloids
Mast cells
Tumoral cells
B cells
Identity
CD274 (PD-L1)
CSF1R
Average Expression
Percent Expressed
Cell types
B cells
Tumoral cells
Mast cells
Myeloids
Stromal cells
T cells
UMAP_1
UMAP_2
F
CD274 (PD-L1)
CSF1R

Figure S11. Evidence of expression of the transcripts of CSF1R and PD-L1 in the myeloid compartment of human tumor samples. Analysis of published scRNA-sequencing experiments of cohorts of 31 patients with melanoma tumors (**A-C**) and 23 patients with colorectal cancer (**D-F**). **A.** The dot plot represents the percentage of each indicated cell type expressing CD274 (PD-L1) and CSF1R transcripts and the intensity of such expression. **B.** The UMAP represents a dimension-reduced projection of different color-coded cell types. **C.** The UMAPs highlight the intensity of expression of the targets of interest by the different cell types. **D-F.** As described in A-C.
